# Artificial intelligence and blockchain implementation in supply chains: a pathway to sustainability and data monetisation?

**DOI:** 10.1007/s10479-022-04785-2

**Published:** 2022-06-21

**Authors:** Naoum Tsolakis, Roman Schumacher, Manoj Dora, Mukesh Kumar

**Affiliations:** 1 Centre for International Manufacturing, Institute for Manufacturing (IfM), Department of Engineering, School of Technology, University of Cambridge, Cambridge, CB3 0FS UK; 2 Department of Supply Chain Management, School of Economics and Business Administration, International Hellenic University, 570 01 Thermi, Greece; 3 Industrial Resilience Research Group, Institute for Manufacturing (IfM), Department of Engineering, School of Technology, University of Cambridge, Cambridge, CB3 0FS UK; 4 Brunel Business School, Brunel University London, London, UB8 3PH UK

**Keywords:** Supply chain digitalisation, Artificial intelligence, Blockchain technology, Sustainability, Data monetisation, Fish supply networks

## Abstract

Digitalisation is expected to transform end-to-end supply chain operations by leveraging the technical capabilities of advanced technology applications. Notwithstanding the operations-wise merits associated with the implementation of digital technologies, individually, their combined effect has been overlooked owing to limited real-world evidence. In this regard, this research explores the joint implementation of Artificial Intelligence (AI) and Blockchain Technology (BCT) in supply chains for extending operations performance boundaries and fostering sustainable development and data monetisation. Specifically, this study empirically studied the tuna fish supply chain in Thailand to identify respective end-to-end operations, observe material and data-handling processes, and envision the implementation of AI and BCT. Therefore, we first mapped the business processes and the system-level interactions to understand the governing material, data, and information flows that could be facilitated through the combined implementation of AI and BCT in the respective supply chain. The mapping results illustrate the central role of AI and BCT in digital supply chains’ management, while the associated sustainability and data monetisation impact depends on the parameters and objectives set by the involved system stakeholders. Afterwards, we proposed a unified framework that captures the key data elements that need to be digitally handled in AI and BCT enabled food supply chains for driving value delivery. Overall, the empirically-driven modelling approach is anticipated to support academics and practitioners’ decision-making in studying and introducing digital interventions toward sustainability and data monetisation.

## Introduction

The digitalisation of business operations is critical for traditional enterprises to compete in the digital economy era (Weill & Woerner, 2018). In this regard, the adoption of advanced systems and applications such as the Internet of Things (IoT), Blockchain Technology (BCT), Cloud Computing, Data Analytics and Artificial Intelligence (AI), in tandem with the development and maturity of relevant digital skills and capabilities, are fundamental for the digital transformation of businesses (Akter et al., 2022). Individually, each digital application presents specific technical merits and differently enacts upon data and information. In the data analysis field, AI is “*a system’s ability to interpret external data correctly, to learn from such data, and to use those learnings to achieve specific goals and tasks through flexible adaptation*” (Haenlein & Kaplan, 2019, p.1). The market value of AI in the food and beverages industry is expected to reach US$29.94 billion by 2026, at a CAGR of 45.8% (ResearchAndMarkets, 2021). In the hardware domain, BCT is a distributed ledger enabling secure data sharing for better visibility and transparency in supply chains (Kamble et al., 2019). The global market size of BCT in agriculture and the food sector is projected to reach US$948 million by 2025, at a CAGR of 48.1% (MarketsAndMarkets, 2020).

Notwithstanding the advantages of digital systems and applications, their interconnectivity could help overcome inherent limitations and unleash additional technical capabilities, harnessing productivity benefits and fostering corporate growth (Akter et al., 2022). Indicatively, AI, the most impactful application in manufacturing at the beginning of the 21st Century, typically leverages centralised computing and data storage infrastructure to explore (continuous) data flows for (real-time) decision-making (Nasar et al., 2020). However, AI systems encounter complicated issues such as data security and interoperability, adversarial attacks, morality, and ethics (Awad et al., 2018). To a greater extent, AI is considered a “black box”, and scepticism pertains to the use of emanating analysis results in critical decision-making. In a similar vein, BCT, as a standalone application, ensures decentralised data and decision storage across supply chains but cannot be used to analyse data and generate intelligence for informing the decision-making process (Salah et al., 2019). However, within the dynamic global business landscape, interactive decision-making based on analysis and interpretation of real-time and reliable data and information stemming from multiple diverse sources is becoming prominent (Toorajipour et al., 2021) for a range of purposes such as avoiding risks and product recalls. In this regard, the combined implementation of AI and BCT and other cutting-edge technologies (e.g., sensor-driven automation for gathering shop floor data) is catalytic to complement technical capabilities, create actual business value and enable competitiveness (Hughes et al., 2022).

Research evidence recognises that BCT can augment the implementation of AI in both upstream and downstream supply chain operations (Grover et al., 2022). Specifically, BCT is considered an adequate digital application to ensure interpretable and trustworthy AI in real-world settings via ascertaining data security, privacy, reliability, usability, dependability, performance, and governance (Nasar et al., 2020). Equivalently, AI-based solutions could support BCT implementations to redefine industrial operations across multiple fronts, including: (i) being more proactive; (ii) enabling autonomous processes; (iii) allowing personalised services; and (iv) transitioning to production planning based on predictions (Toorajipour et al., 2021). In addition, leveraging the synergistic technical capabilities of multiple digital systems and applications such as AI and BCT helps pursue Sustainable Development Goals (Del Río Castro et al., 2021). Collaborative digital ecosystems promote sustainable supply chains performance via enabling the improved management of resources, supporting waste monitoring and management, reducing energy consumption, and informing the development and diffusion of sustainable financing instruments (Belhadi et al., 2021; Kumar et al., 2022).

The combined implementation of data-driven digital technologies is particularly valuable in a food supply chain context due to the increasing demand for verifiable transparency and traceability evidence regarding product safety and quality (Aung & Chang, 2014). Specifically, integrating AI and BCT could expand the data gathering, interoperability and analysis capabilities across end-to-end echelons of operations to enable supply network security, cost-competitive resiliency, and sustainability, thus ultimately leading to enhanced consumers’ trust (Bechtsis et al., 2021). Major retailers have already implemented BCT, namely: (i) ‘Walmart’ for tracing agricultural products in the U.S. and pork in China; (ii) ‘Carrefour’ for tracking milk supply chains; and (iii) ‘Alibaba’ for addressing food fraud (Kshetri, 2018). At an institutional level, the U.S. Food and Drug Administration pilots an AI-driven blockchain implementation to dynamically assess foodborne illness risks from imported food supplies and enhance end-to-end tracking and tracing of goods (Mearian, 2019).

In terms of international trade, AI and BCT could help unleash benefits for the involved system stakeholders. For example, the European Union (EU) is Thailand’s third-largest export destination for seafood products after the U.S. and Japan, accounting for 10% of the total exports. In fact, in 2017, the value of Thailand’s total exports of fishery products was worth about US$5.93 billion (Kishimoto, 2019). To this effect, the potential exclusion of Thailand from exporting seafood products to the EU is associated with significant economic and social ramifications. In April 2015, Thailand was issued a ‘yellow card’ for violating EU standards concerning fisheries management. The ‘yellow card’ is a formal notice signalling that the exporting country does not take sufficient measures to tackle illegal fishing. In case appropriate measures are not subsequently implemented, the country concerned will be excluded from trade operations with the EU (European Commission, 2015); Thailand was delisted in 2019 (IUU Watch, 2020). Illegal overfishing and fishing of endangered species are two major sustainability concerns that the Thai fish industry must tackle to remain delisted (European Commission, 2015). Illegal fishing is not a phenomenon exclusively concerning Thailand but instead constitutes a global issue requiring immediate and decisive action. On the business side, illegal fishing is a severe externality that threatens the entire industry (Ryan et al., 2014). From a social viewpoint, as a typical example of an economic common good, fish is ‘rivalrous’ (i.e., the consumption from one person excludes the consumption of another person) and non-excludable (i.e., people who have not paid for it cannot be prevented from having access to it). Overfished species have tripled in the last half-century, and one-third of all fish stocks are no longer at their biologically sustainable levels (FAO, 2020). The sustainability implications extend beyond the purely maritime ecosystem. Billions of people depend on fish as a source of protein, and millions live from fishing (WWF, 2020).

A central problem in the legal action against illicit fishing activities is the lack of transparency across the supply chain. Globally, many fishers do not comply with fisheries legislation and can sell their catch on the market without proof of compliance (Macfadyen et al., 2019). Responding to the transparency challenge, AI and BCT have proven effective tools to reduce information asymmetry and increase transparency across supply chains (Bumblauskas et al., 2020; Ebinger & Omondi, 2020). However, multiple and diversified data archetypes often exist in end-to-end supply chains. Key challenges in implementing these technologies are often related to limited processing capabilities of unstructured, incomplete, and sometimes inaccurate data (Choi et al., 2020). Furthermore, a lack of systems thinking in supply chain participants can lead to significant challenges in implementing such technologies in complex industrial contexts (Camaréna, 2020). To a greater extent, AI and BCT implementations can leverage data to inform decision-making in business processes and provide data-driven products and services. Therefore, the concept of ‘data monetisation’ emerges, i.e., “*… using data from an organization to generate profit*” (Faroukhi et al., 2020, p.1). Data monetisation related to the traceability awareness of consumers and production costs of suppliers and manufacturers has been investigated (Fan et al., 2022). Research findings indicate that consumers aware of traceability are willing to pay a higher price in the supply chain, up to a certain threshold where the traceability awareness impact diminishes (Fan et al., 2022).

A key challenge to digital-driven traceability and sustainability in food refers to the lack of appropriate information technology-based tools that can inform the design of supply chains for agility and dynamic change (Klein et al., 2016). Therefore, digital implementations shall emerge as knowledge-based activities (Yu, 2009), emanating from the empirical understanding of complex enterprise phenomena at a conceptual modelling level (Guizzardi et al., 2013). Concerning modelling, methodologies are required to rapidly redesign digital supply chains to respond to the socio-technical and environmental developments whilst ensuring data analysis, information flows, and operational understanding (Fayoumi & Loucopoulos, 2016).

This research aims to explore the interplay among AI, BCT and supply chain operations that could promote sustainability and value delivery, with a particular focus on food networks. We share the view that material flows, data-based transactions and information generation, enabled through AI and BCT, can allow actors across the supply chain to monetise these data (i.e., harness value). To this effect, the objective of this research is to devise a systematic analysis approach for understanding the interplay among digital implementations and the supply chain ecosystem that allows for exploring the associated sustainability and data monetisation opportunities. Therefore, we attempt to address the following research question: *How can the interplay of digital technologies in food supply chains be captured for sustainability and data monetisation*?

To respond to the articulated research query, we first employ business process mapping and a Systems Thinking perspective to capture material, data, and information flows across dynamic supply chain operations. As the implementation of AI and BCT can facilitate the fundamental flows across an end-to-end supply chain, the mapping process focuses on the role of digital technologies. By applying such a mapping approach, this research represents the dynamics and determines the performance of operations in an illustrative tuna manufacturing supply chain. In particular, the business process mapping allows capturing the significant key data elements to be visible (e.g., via BCT infrastructure) and interpretable (e.g., via AI algorithms) to all involved stakeholders. Furthermore, the Systems Thinking perspective provided the possibility to capture the interplay among the corresponding ‘AI-BCT-supply chain’ structural elements and explore the underpinning dynamics.

Akter et al. (2022) stressed the need to investigate the combined use of emerging technologies in digital business transformation for operational excellence and sustainable growth. This research contributes to the Operations Management field by proposing an empirically-driven framework that provides an understanding of the ‘digital technology – supply chain’ system structure and the interplay between these two domains for proactively assessing the emanating operations-wise benefits, responding to a documented gap in the community (Sodhi et al., 2022). Specifically, this research’s findings guide the joint implementation of AI and BCT in supply chains for leveraging these two technologies’ inherent synergic value and inform supply chain managers’ expectations at the initial stage of these emerging technologies’ adoption. To the best of our knowledge, this is one of the first studies that highlight the unique advantages emanating from the combined implementation of AI and BCT and discuss the resulting sustainability and data monetisation gains in fish supply chains.

The remainder of this research is structured as follows. Section 2 overviews the research background on AI and BCT in supply chains by highlighting benefits, implementation challenges and sustainability implications. Section 3 details the underpinning research methodology for designing supply chains based on AI and BCT. Section 4 outlines fish supply chains and essential key data elements, while Sect. 5 elaborates on the case study of the fishery supply chain ecosystem in Thailand. A proposed research framework is inserted in Sect. 6. Conclusions, implications, limitations, and future research avenues are explored in the final Sect. 7.

## Artificial intelligence and blockchain in supply chains

The introduction of AI in supply chain management facilitates the orchestration and optimisation of network operations via: (i) revealing complex behavioural patterns through multifaceted analysis of data (e.g., classification, optimisation, clustering); (ii) perceiving the surrounding environment to inform autonomous activities and proactively address emerging performance and quality issues; (iii) informing supply chain design, simulation and planning; and (iv) enabling negotiation-based collaborative modelling (Toorajipour et al., 2021). Moreover, BCT is an application that enables “*transparent, secure, decentralized ledgers, smart contracts and reliable networks for sustainable supply chain management*” (Kouhizadeh et al., 2021, p.15). Therefore, this research proposes the joined and complementary implementation of AI and BCT to increase supply chain efficiency and sustainability, focusing on the food sector.

In order to identify the extant research on the utilisation of emerging technologies, specifically AI and BCT, in the context of supply chains, we performed a critical literature taxonomy. For this purpose, we conducted a structured Boolean-type keyword search in the Scopus database (Aivazidou et al., 2016). Although we acknowledge that both the Scopus and Web of Science databases cover the vast majority of scientific journals in the fields of business and management, natural sciences and engineering that are relevant to this study (Mongeon & Paul-Hus, 2016), we exclusively used the Scopus database for our literature search queries. We focused on Scopus because it is a widely accepted database for searching and mapping the extant literature (Fahimnia et al., 2019; Pournader et al., 2020). A systematic literature review extends the scope of this research. The Boolean keyword search was conducted using the following combination in the “Article title, Abstract, Keywords” field: {“Artificial Intelligence” AND “Blockchain” AND “Supply Chain”}. The search was further limited to journal articles’ publications written in English. The time horizon of publications was left unrestricted. By the 5th of January 2022, the search returned 36 results. After reading the abstracts, nine papers were excluded as deemed irrelevant to the scope of this research. The remaining 27 results are listed in Table 1.

**Table 1:** Critical taxonomy of the existing research

Study	Supply chain type	Methodology	Technology			Technology scope in supply chains
			AI	BCT	Joint Implementation (AI & BCT)	
Agrawal and Narain (2021)	Not specified	Structural modelling	✓	✓		Digital transformation; Technology adoption
Barczak et al. (2019)	Not specified	Case study	✓	✓		Technology implementation risks
Baz et al. (2022)	Medical	Literature review	✓	✓		Covid-19 pandemic risk management
Bechtsis et al. (2021)	Food	Mixed (Case study & Literature review)			✓	Data monetisation
Cagliano et al. (2021)	Not specified	ANOVA	✓	✓		Technology adoption
Chidepatil et al. (2020)	Waste	Case study			✓	Circular supply chains
Dillenberger et al. (2019)	Not specified	Case study			✓	Logistics optimisation
Dwivedi et al. (2021)	Not specified	Literature review		✓		BCT – Internet of Things integration
Ebinger and Omondi (2020)	Not specified	Literature review	✓	✓		Supply chain transparency
Eluubek kyzy et al. (2021)	Agriculture	Technical framework		✓		Data monetisation
Ethirajan et al. (2020)	Manufacturing	Case study	✓	✓		Operational optimisation of supply chains
Hartley and Sawaya (2019)	Manufacturing and services (multiple)	Case study	✓	✓		Assessment of technology potential
Hopkins (2021)	Not specified	Survey	✓	✓		Supply chain innovation
Kale et al. (2020)	Healthcare	Conceptualisation		✓		Supply chain transparency and efficiency
Kamble et al. (2022)	Manufacturing	Literature review	✓	✓		Sustainability
Liu et al. (2021)	Food	Literature review	✓	✓		Key applications
Luo and Choi (2022)	Not specified	Literature review	✓	✓		Literature categorisation
Mehta et al. (2021)	Oil and gas	System modelling		✓		Data security
Nandi et al. (2021)	Not specified	Conceptualisation	✓	✓		Sustainability
Probst (2020)	Fish	Literature review	✓	✓		Supply chain transparency
Reyes et al. (2020)	Not specified	Literature review	✓	✓		Technology implementation
Rodríguez-Espínola et al. (2020)	Humanitarian operations	Case study			✓	Disaster management
Sgantzos and Grigg (2019)	Not specified	Literature review			✓	Data integrity
Singh and Chaddah (2021)	Pharmaceutical	Survey		✓		Supply chain security
Sobb et al. (2020)	Military	Literature review	✓	✓		Cyber security
Stanislawski and Szymonik (2021)	Multiple	Survey	✓	✓		Technology impact on market positioning
Zhou et al. (2020)	Not specified	Conceptualisation; Literature review	✓			Fraud detection

*AI* Artificial intelligence; *BCT* Blockchain technology

### Artificial intelligence

As information becomes increasingly available throughout global supply chains, so do the expectations for AI’s use of this information (Sanders et al., 2019). In fact, a study from McKinsey estimated that AI analytics could add around US$13 trillion (or 16%) to annual global GDP by 2030, while essential supply chain relevant operations (e.g., logistics, retailing) could be benefited the most (Bughin et al., 2018). Accordingly, supply chains’ efficiency and productivity are set to increase significantly due to the use of AI over the next decade. In the supply chain spectrum, the introduction of AI implementations adds value by: (i) facilitating supply network design and reconfiguration through vetting and classifying potential stakeholders (e.g., alternative suppliers), facilities and technologies (Govindan et al., 2017); (ii) analysing big data for explaining and assessing risks thus promoting supply chain resilience (Papadopoulos et al., 2017); (iii) supporting near real-time, automated and optimal decision-making via analysing large amounts of data from diverse sources (e.g., web, social media, information systems of involved supply chain actors) to address uncertainty and demand volatility (Baryannis et al., 2019); and (iv) enabling learning, reasoning and self-correction of supply chain operations whilst promoting validation of information for particular purposes such as contracting (Shen et al., 2019).

Contemporarily, creating sustainable global supply chains has emerged as one of the most urgent yet unresolved industrial challenges (Dauvergne, 2020). End-to-end global supply chain operations profoundly impact sustainability (Carter & Washispack, 2018). Most negative environmental impacts do not emerge from direct manufacturing operations but from end-to-end supply chain operations that involve sourcing, distribution, production, and logistics (Sanders et al., 2019). Although many research scholars propagate AI as ground-breaking for the design of ‘green’ supply chains, others see the implementation of AI in supply chains as an acceleration of existing negative influences on sustainability.

This research acknowledges that the discussion on the advantages and disadvantages of AI for sustainability can be conducted on many levels. To this end, Table 2 provides an overview of frequently identified sustainability benefits and challenges stemming from the implementation of AI in supply chains.

**Table 2:** Literature taxonomy of Artificial Intelligence implementation for supply chain sustainability

Author(s)	AI aim	AI implementation benefits	AI implementation challenges	Study nature	Study methodology
Baryannis et al. (2019)	Identification, assessment, mitigation and monitoring of SC risks	Enables accelerated and adaptive decision making based on large datasets Provides automated decision-making, predictive and learning capabilities Enables increased SC visibility	N.A.–N.S	Ql	Literature review
Camaréna (2020)	AI for transitioning food systems	Supports increase in productivity (production optimisation) Ensures automation of seeding, crop yield, harvesting, etc Informs decision-making Enables machine learning for detecting the decomposition rate of vegetables Enables machine learning and machine vision to detect parasites on fish Helps detect environmental pollution	Legal and ethical issues of responsibility and liability (e.g., through automated decision-making) Exacerbation of tensions between social and technical engineering Jeopardising of data confidentiality (e.g., by hacking by competitors and foreign powers) Privacy rights infringements of farmers by governments through applying AI for SC traceability Lack of required systems thinking by participants in the food system	Ql	Literature review
Chidepatil et al. (2020)	Blockchain and sensor-driven AI for transforming the circular economy	Facilitates information sharing among SC members Enables smart contracts in the procurement processes between SC members	Lack of reliable information about availability, quantity, quality, and suitability	Ql	Case study
Cubric (2020)	Drivers and barriers to AI adoption in business and management	Supports increased productivity Promotes cost reductions (e.g., reduced human errors, equipment cost, reduction of human labour, etc.) Supports decision-making (e.g., informed decisions, more accurate forecasting, etc.) Outlines more sustainable processes to fit consumer demand (e.g., agriculture)	Labelling/structuring data can be costly Support of infrastructure is necessary Lack of training data may lead to reduced performance of AI algorithms Data may be unstructured and thus challenging to share Data confidentiality issues Generalisability of data may not be possible Difficulties in reusing AI models for different purposes Lack of knowledge about the benefits of AI for specific problems	Ql	Literature review
Dauvergne (2020)	Environmental impact of AI	Promotes corporate efficiency and productivity gains Enhances energy efficiency of transnational corporations, e.g., automation of cooling systems with AI Promotes efficiency and reliability gains of renewable energies, e.g., forecasting weather, fine-tuning energy storage, etc Helps reduce fuel consumption by increasing efficiency in production and logistics Supports programs to cut costs of packaging and shipping	Negative environmental impact Increases in consumption and production outweigh positive effects on corporate social responsibility Enhanced social inequality Accelerated natural resources extraction Racial and ethnic profiling of customers	Ql	Literature review
Di Vaio et al. (2020)	Impact of AI on agri-food SCs	Promotes testing concerning food safety at every stage of the SC Promotes increases in efficiency and productivity Enables food savings, improves the hygiene of production sites, and helps clean up production equipment quickly Enables food sorting (e.g., optical sensor-based solutions with machine learning capabilities) Helps ensure hygiene standards (e.g., use of cameras to monitor the compliance with hygiene standards) Helps the use of drones to assess the ripening status of crops	Enhanced social inequality Privacy issues due to AI application data Required significant changes in business models	Ql	Literature review
Ebinger and Omondi (2020)	Transparency in sustainable SCs	Enables traceability and tracking through the collection and processing of big data for end-to-end SC visibility Fosters cooperation and partner selection via real-time information sharing for decision-making processes Promotes governance via end-to-end SC data collection and processing Assists in strategic and operational risk assessment via data analytics	Individualisation of digital solutions in companies via using internal data architecture Lack of standardised information leading to difficulties in choosing appropriate solutions to increase transparency Many approaches used are still in the trial phase and offer only limited solutions	Ql	Literature review
Min (2010)	Support of decision-making: strategic/ operational/ tactical	Provides a decision support tool that can help firms connect with customers, suppliers and SC partners Facilitates information exchange among SC entities Facilitates understanding of SC dynamics Supports the logistics outsourcing or contract manufacturing decisions Supports supplier selection Facilitates real-time pricing and reverse auctioning involving SC partners	Solutions may be too costly Difficult to produce solutions due to the complexity and ill-structured nature of the problem Relative youth and broad spectrum of the SC management discipline AI solutions may be too difficult for decision-makers to comprehend Difficulties in implementation for handling risks in cross-functional or cross-border contexts due to knowledge acquisition bottlenecks	Ql	Literature review
Mota et al. (2015)	AI for economic, environmental and social design and planning	Helps improve all sustainability dimensions (i.e., economic, environmental, social) Promotes cost reductions and mitigation of environmental hazards through modelling (e.g., reducing warehouses) Helps increase social sustainability with a slight compromise of economic performance	Indicators for sustainability in the dimensions considered for this work are imperfect/can be significantly improved No possibility to evaluate the social dimension of an SC per se Consideration of only a single SC	Qn	Generic multi-objective mathematical programming model
Orji and Wei (2015)	Fuzzy logic and system dynamics for sustainable supplier selection	Helps in the modelling of supplier selection criteria for decision support	No spatial and temporal considerations regarding decisions Criteria and alternatives in the model are fixed	Qn	Fuzzy logic and system dynamics
Sanders et al. (2019)	AI for sustainable SCs	Helps improve demand management Promotes increased operational productivity Promotes increased operational efficiency Enables increased SC transparency, traceability and security Supports decision support (e.g., pricing)	Infliction of severe economic and social cost Creation of biases and discrimination through machine learning applications Potential failure of AI algorithms to detect misinformation	Ql	Literature review
Ting et al. (2014)	Quality assurance in food SCs	Supports decision-making Helps create and transfer quality relevant information Promotes inspection cost reductions Promotes quality level enhancements Promotes increased customer satisfaction Enhances SC visibility	Increasing costs for transportation Requirement of significant time to identify dominant association rules by computational methods	Qn	Association mining

*AI* Artificial intelligence; *SC* Supply chain; *Ql.* Qualitative; *Qn.* Quantitative

#### Sustainability benefits

The implementation of AI is recognised for enabling various operational benefits, such as increasing productivity and efficiency (Camaréna, 2020; Cubric, 2020; Di Vaio et al., 2020; Sanders et al., 2019), thus leading to increased economic sustainability. Indicatively, AI is used to optimise the harvesting and processing of crops, e.g., by using drones with cameras and machine learning algorithms to determine the decomposition rate of vegetables (Camaréna, 2020). Additionally, AI supported by other constituent technologies facilitates the sorting of food supplies whilst continuously monitoring the hygiene level across operations (Di Vaio et al., 2020). Similar benefits could also be achievable for the fishing industry on multiple fronts, from monitoring fish harvesting and downstream industrial processing to ensuring transparency and traceability across international trade operations (Tsolakis et al., 2021). AI can further help reduce operational costs related to human errors, labour, and equipment (Cubric, 2020) or costs related to fuel consumption for production and transportation (Dauvergne, 2020). Except for the benefits at an (internal) operations level, AI has also been identified to provide benefits for the end-to-end supply chain management. Such benefits include improved customer demand management and forecasting (Cubric, 2020), increased supply chain transparency (Ebinger & Omondi, 2020), decision-support on the pricing of products (Min, 2010; Sanders et al., 2019), and information sharing among supply chain stakeholders (Chidepatil et al., 2020).

From a social sustainability perspective, transparency is one of the key benefits that AI can deliver, particularly downstream a supply chain. For example, AI can inform customers about making more conscious purchasing decisions towards responsibly sourced and produced goods due to the advanced data processing capabilities that allow tracing the upstream supply chain to the raw materials stage (Chidepatil et al., 2020). Furthermore, AI-driven applications can contribute to social welfare; for example, socially assistive robots can decrease the workload of caregivers and enhance the well-being of the elderly population by enabling mobility, social contacts, and cognitive support (Cubric, 2020).

Moreover, the implementation of AI can help promote environmental sustainability. In the energy domain, AI can contribute to reducing fuel consumption by increasing efficiency in energy conversion and logistics. Furthermore, AI can provide efficiency and reliability gains of renewable energies, e.g., by increasing weather forecast accuracy and fine-tuning energy storage (Dauvergne, 2020).

#### Implementation challenges

The challenges for AI implementation comprise technical, ethical, legal, managerial, and socio-economic considerations. A major technical challenge for adopting AI in business operations relates to the availability and use of data. Data available to firms is often unstructured and difficult to share between the supply chain members. Structuring this data can be very costly. Furthermore, the data used for a specific case might not be generalisable (Cubric, 2020). For example, problems can arise by (small) datasets, which do not accurately reflect reality, or by overfitting the AI algorithm to the training data set. On the other end, a lack of training data may lead to reduced performance of the elaborated AI algorithms (Cubric, 2020). In addition, a lack of standardisation of information can lead to difficulties in choosing the right AI solution. There is a trend toward individualising companies’ digital solutions via internal data architecture (Ebinger & Omondi, 2020).

Another challenge arising with the use of data for AI is the possibility of privacy rights infringement. For example, using AI-enabled traceability of food products across a supply chain by governments or competitors can inflict infringement on farmers’ privacy rights (Leone, 2017). Furthermore, project datasets often contain confidential information, leading to significant technical barriers to adopting AI-driven solutions in industrial applications. Additionally, the application of AI may impose social problems with ethnical and racial profiling. Indicatively, facial recognition algorithms in a shopping mall in St. Petersburg already profile customers by age, ethnicity, and gender (Dauvergne, 2020), thus raising privacy concerns.

In addition to ethical and legal challenges related to data, the implementation of AI encounters significant barriers owing to the relative youth and broad spectrum of the discipline (Min, 2010). Due to the early development and application stage, many AI-based solutions are demonstrated only in pilot/trial demonstrators and offer limited practical solutions (Ebinger & Omondi, 2020). Accordingly, there is often a lack of managerial awareness about the implementation benefits of AI in corporations (Cubric, 2020). In this regard, any AI-based solutions might be complicated for decision-makers to comprehend (Min, 2010).

Lastly, despite the benefits stemming from the implementation of AI in an industrial context, a range of significant social risks is involved (Di Vaio et al., 2020). As technologies like autonomous driving are developing fast, unemployment issues for professional truck drivers might arise in the long term (Sanders et al., 2019). The possibility of AI-driven solutions replacing human labour will exacerbate social and technical engineering tensions (Camaréna, 2020).

### Blockchain technology

Blockchain is a type of database that stores data in blocks distributed across a network of operations in a decentralised manner. New data and information added by an actor across a supply chain would be shared with the other actors almost instantly. The data blocks are interlinked with a hash (i.e., cryptographic ‘fingerprint’) of all previous blocks.

Considering the food sector, blockchain implementation in supply chains is reasonably nascent but growing since the technology enables digital ‘passports’ to physical products. For example, Project Provenance Ltd uses the Ethereum blockchain to help producers prove the authenticity and origin of yellowfin and skipjack tuna in fish supply chains in Indonesia (Provenance, 2016). Furthermore, Intel piloted blockchain in seafood supply chains via using Hyperledger Sawtooth (del Castillo, 2017). In addition, the World Wide Fund for Nature also piloted the use of blockchain to trace fish from its origin, specifically focusing on the tuna industry of the Pacific Islands (WWF, 2018). Lastly, FishCoin developed a blockchain traceability platform for fisheries and modelled incentives for participants to share data in return for tokens (Fishcoin, 2018).

#### Sustainability benefits

Blockchain creates opportunities for improving national sector-specific supply chains to drive competitiveness, trade, and the triple-helix of sustainability (Kimani et al., 2020). In terms of economic sustainability, for example, the World Trade Organisation expects that the removal of trade barriers owing to the implementation of blockchain could result in new trade operations of more than US$1 trillion during the next decade (Ganne, 2018). The EU recognises the enabling role of BCT to supply chains and international trade, particularly in terms of: (i) customs facilitation; (ii) greater inclusivity of small and medium enterprises; (iii) sustainable trade realisation; and (iv) accelerated clearance processes at borders and terminals thus minimising waiting times (Copigneaux et al., 2020).

In the social sustainability domain, BCT enables data security and immutability owing to its technical characteristics that prevent modifying the shared data blocks without ‘breaking’ the chain (Babich & Hilary, 2020). In this regard, the implementation of BCT in a supply network allows increased levels of traceability hence enabling importers’ judgement over the responsible sourcing and processing of products (Copigneaux et al., 2020).

Experts also share the view that BCT fosters environmental sustainability in international supply chain operations. Particularly, blockchain allows the digitalisation of trade documentation, thus leading to less use of paper (Copigneaux et al., 2020). To a greater extent, BCT can lead to fewer carbon emissions, e.g., by reducing the fuel consumption of freight vehicles waiting at the borders (Copigneaux et al., 2020).

#### Implementation challenges

The practical use of BCT entails several organisational, technical, and operational challenges and barriers that need to be overcome, including: (i) data storage capacity and scalability; (ii) security weaknesses and threats; (iii) anonymity and data privacy; (iv) legal issues; and (v) consensus among blockchain participants (Reyna et al., 2018).

From an organisational perspective, inter-organisational and intra-organisational barriers to adoption exist, mainly including financial resources, organisational readiness, legal and regulatory compliance, and standardisation (Dutta et al., 2020). Most importantly, the limited awareness of professionals about blockchain hinders its adoption in supply chains (Kamble et al., 2019). To a greater extent, behavioural expectations and limited trust among multiple stakeholders in a supply network can imperil BCT implementation initiatives (Queiroz & Fosso Wamba, 2019).

From a technical viewpoint, challenges for implementing BCT in supply chain operations refer to scalability, interoperability, product governance, and latency (Dutta et al., 2020). Considering the global operations in modern supply chains and the inclusion of multiple stakeholders, from tier level suppliers to end consumers, the challenges of blockchain integration in supply chain operations are pivotal (Dutta et al., 2020). In addition, as BCT ensures secure data transactions, the size of the data blocks is a key factor impacting the performance and efficiency of such a digital platform (Li et al., 2019).

At an operational level, technological systems’ compatibility, adaptability, standardisation, and expandability are the main challenges to BCT implementation across supply networks (Sharma et al., 2018; Wang et al., 2019). Specifically, in the food sector, the modus operandi typically requires myopic traceability involving only directly linked actors. However, the increased frequency of disruptions, food scandals, and product recalls have implied the necessity for establishing chain visibility and traceability, possibly enabled via BCT, to foster resiliency (Katsaliaki et al., 2021) ultimately. Nevertheless, this need to apply “*diligent and time-consuming bookkeeping and labeling by all members of a facility*” is challenging, particularly in the food sector (Bumblauskas et al., 2020, p.3). An engineering challenge is also the energy supply and storage capacity of such devices (e.g., sensors’ battery life longevity, servers’ energy requirements) to enable near real-time data gathering.

### Artificial intelligence and blockchain technology integration

Considering that AI and blockchain are relatively nascent, real-world joint implementations of these technologies are rare. Nevertheless, few scientific works theoretically discuss the advantages and disadvantages of respective integrated solutions.

First, the integrated implementation of AI and BCT serves to overcome inherent limitations characterising each of these individual technologies. On the one end, in the application of AI, trustworthiness, explainability, and lack of sufficient data and privacy issues are often significant barriers to implementation. On the other end, blockchain demonstrates weaknesses in terms of scalability and efficiency. Therefore, the integrated implementation of both technologies can compensate for the individual weaknesses in a complementary manner (Dinh & Thai, 2018; Rodríguez-Espíndola et al., 2020).

AI can provide efficient data-driven decision support for various business problems. However, complications in the flow of data and information across a supply chain can negatively affect the performance of AI algorithms which require accurate, reliable, and timely input (Rodríguez-Espíndola et al., 2020). Blockchain can ensure such flows by serving as a platform for managing and sharing data and information from multiple sources whilst ensuring the traceability and accountability of the flows (Dinh & Thai, 2018; Rodríguez-Espíndola et al., 2020).

Data that is systematically stored on enterprise blockchains can eliminate a vast amount of time in pre-processing and can inform supply chain decision-making. Dillenberger et al. (2019) reported multiple real-world use cases around the IBM Blockchain Platform. For example, IBM’s data science tool IBM Watson Studio combined AI and BCT to optimise logistics processes, i.e., to predict potential shipping delays using historical shipping, weather, and location data. BCT enabled access to relevant structured data from the supply chain, while AI could predict the delays based on this data. Furthermore, using BCT as a decentralised platform to share data and information enables a more efficient allocation of resources in need within the platform, where the prioritisation of stakeholders or destinations can be supported by AI (Rodríguez-Espíndola et al., 2020). In addition, AI-driven decisions may lack interpretability, leading to ambiguity and lack of implementation of analysis algorithms as decisions cannot be trusted or verified by humans. Blockchain can help overcome this challenge by ensuring the traceability of data processing and decisions throughout a supply network (Dinh & Thai, 2018).

Vice versa, AI can benefit blockchain (Dinh & Thai, 2018). For example, AI can intelligently maintain BCT, optimise and ensure the quality and robustness of smart contracts, and automate malicious behaviour detection in the blockchain (Zheng et al., 2019). More precisely, AI-powered algorithms can detect failures and performance bottlenecks in blockchain systems, and they can further be employed to detect bugs in smart contracts (Zheng et al., 2019). Improving the security of the blockchain is significant to maintaining trust in the technology. Prior cyber-attacks on blockchain-based systems, for example, the Decentralised Autonomous Organisation (DAO) or bitcoin gold, led to significant economic and reputational damage. In the case of the DAO, hackers stole cryptocurrency worth about US$50 million by exploiting the vulnerabilities of a smart contract that was used on top of a blockchain platform (De, 2017; Dinh & Thai, 2018). Analytics using blockchain data can be used to identify fraudulent or malicious behaviour of the respective users (Zheng et al., 2019).

In addition, AI can enable the storage of high quality and reliable information that can be shared among stakeholders. A real-world example was presented by Chidepatil et al. (2020), who investigated a case on plastic feedstock in collaboration with the Radical Innovations Group Finland, a technology provider for Circular Economy solutions. Currently, chemical feedstocks are preferred over recycled plastic feedstocks by manufacturers, mainly due to a lack of information about the quality and availability of recycled plastics. The study results showed that AI could help improve the information about recycled plastics in circular supply chains. AI can train a system to recognise and segregate waste properly and generate relevant information about the grade of the recycled material. The information is stored and shared on a blockchain, thus improving the transparency of information about supply chain relevant indicators, such as quality, suitability and availability of plastic feedstock, incentivising stakeholders to move from virgin polymers to recycled materials.

The combined implementation of AI and BCT in a supply chain context can help overcome inherent technical limitations. Thereafter, leveraging the synergistic action of these digital technologies helps apply a dynamic decision-making process and foster operational improvements to harness triple-helix sustainability benefits from preventing resources overexploitation to tackling fraudulent incidents, eliminating product recalls, and promoting gender and cultural equality. Ultimately, the operational improvements driven by data-centric decision-making directly promote data monetisation through cost reductions and profit generation (Bechtsis et al., 2021). Following the notion pertinent to the healthcare sector, the gathered (anonymised) data could also be used as a source of income (Kamel Boulos et al., 2018).

### Literature remarks

Our literature taxonomy revealed limited evidence on critical decision-making enabled by the secure management of trustworthy data streams via BCT whilst ensuring real-time data processing via AI implementations (Bechtsis et al., 2021). In the existing supply chain management research on AI and BCT, the two technologies are mainly viewed in isolation, and the benefits of their combined use remain largely unexplored. Furthermore, most studies focus on technical aspects of these technologies per se, whilst the supply chain implications are often only superficially discussed.

The majority of extant studies are literature reviews that discuss AI and BCT under the umbrella of digital technologies; case studies that explore the benefits of jointly implementing the two technologies for different purposes such as supply chain optimisation or waste prevention are scant. Especially, our literature review showed that only five works discuss the joint implementation of BCT and AI in the context of supply chain management. It becomes evident that the nascent area of sustainability, coupled with transparency and traceability as thematically related sub-areas, is gaining importance to motivate consumers’ trust (Shen et al., 2022). Nevertheless, the food sector is still under-researched as a safety-critical and sustainability-relevant area. We recognise this research gap and argue that the joint implementation of AI and BCT has the potential to address the problem of data and information accessibility in food supply chains to promote operational efficiency, sustainability, and value delivery. In this respect, this research proposes a framework for the integrated implementation of AI and BCT in food supply chains as a pathway to the triple-helix of sustainability and data monetisation.

## Methodology

Considering that this research shall develop a practical framework for implementing AI and blockchain in supply chains, the object of scrutiny has to be a real-world case study (Gibbert et al., 2008). The theoretical lens and the research approach based on which this study was conducted are described in the following sub-sections.

### Theoretical lens

In the complex and dynamically changing global business landscape, to assess the digital transformation of supply chains for sustainability and data monetisation, this research adopted the lens of Proudlove et al. (2017). The latter described two pertinent stages: (i) conceptualisation of the business processes along with the material, data, and information flows; and (ii) coding of the conceptual model for enabling real-time simulations to inform potential structures of the underpinning flows, assess alternative scenarios and inspire policy debate.

First, an “activity-based” approach is required to map supply chain operations as building blocks and ensure consistency in the understanding and interpretation of processes and flows (e.g., material, data, information) among all stakeholders (Holweg et al., 2018). In this regard, we adopted the principles of the Business Process Model and Notation (BPMN), which is a formalised modelling language for conceptually mapping internal business procedures in a standardised manner (OMG, 2014).

Second, owing to the dynamic nature of food products’ characteristics and the pillars of sustainability and the associated performance, managers shall be able to consider supply chain operations over time (Sterman, 2000). To this effect, we used Systems Thinking to comprehend the underpinning interactive relations of such a complex system (Forrester, 1961), and we complementarily leveraged System Dynamics to develop a time-dependent view of its behaviour (Machuca, 1998). System Dynamics is a method that has been proven successful in policy-making, strategy decision-making and scenario planning (Srai et al., 2022) and could be employed to model the feedback loops that determine the dynamic behaviour of digitally enabled supply chain operations. The role of System Dynamics as a structural theory to explain, analyse and understand operations phenomena is well recognised (Größler et al., 2008).

The use of System Dynamics as the logic of enquiry in assessing alternative policy scenarios over the environmental and economic sustainability dimensions of food supply chains has been widely applied in the extant literature, such as in the case of water footprint in the UK poultry sector (Tsolakis et al., 2018). Thereafter, System Dynamics-based causal-loop diagrams can be interpreted into simulation models based on stock-and-flow diagrams to: determine and approximate material inventories, identify key data elements and information sources, analyse model parameters, assess the impact of alternative supply chain structures and operations (enabled by AI and BCT joint implementations), and articulate a portfolio of alternative material, data, and information flows.

**Fig. 1:**
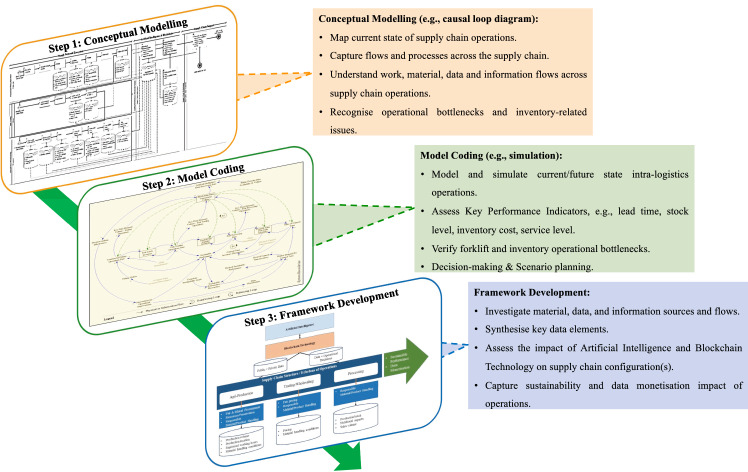
Methodological lens for conceptualising Artificial Intelligent and blockchain technology implementations in supply chains

Ultimately, the integration of these approaches, in terms of conceptual modelling and simulation structuring, leads to articulating a framework for supply chain ecosystems that aim toward sustainability and data monetisation enabled by AI and BCT implementations (Fig. 1).

### Research approach

**Fig. 2:**
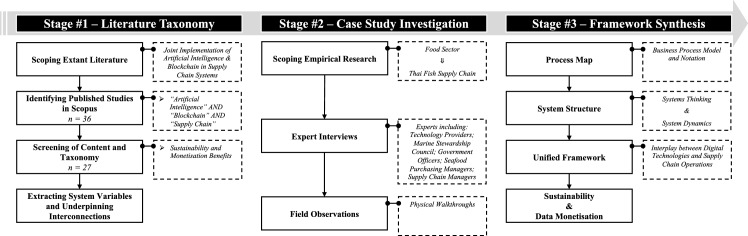
Flowchart of the research methodology

As this research aims to propose a practical framework emanating from theoretical foundations, a case study research approach was adopted (Yin, 2003). In this regard, the case of the Thai fishery industry was selected as an appropriate paradigm for the potential joint implementation of AI and BCT due to the need to tackle challenges pertinent to the Sustainable Development Goals of the United Nations and improve the export outlook of this Thai industry (Tsolakis et al., 2021). Figure 2 illustrates the methodology process flow that was applied in this research.

#### Empirical evidence

In order to identify supply chain operations, pertinent key data elements, and material and information flows necessary for exploring the behaviour of a system combining AI and BCT, expert interviews and physical walkthroughs were conducted in Thailand, as detailed in Table 3. In particular, thirteen open-ended interviews with experts in the Thai fish industry and three physical walkthroughs in related multinational organisations were conducted to map the respective supply chain processes and identify the potential for joint implementation of AI and BCT. The selected informants covered the main stakeholders in the Thai fish industry ecosystem and are classified into seven key categories of actors, including fishermen, traders, processors, wholesalers, technology providers, certification organisations and the government. The number of engaged experts is deemed sufficient as it enables to holistically collect information through different perspectives such as processes, technical, social, and legal issues. The triangulation of data available from different experts (along with the used secondary evidence) within the same system helped address bias phenomena stemming from the interview process, ensuring the quality of the research findings (Yin, 2009). The protocol used to conduct the semi-structured interviews is inserted in Appendix 1.

**Table 3:** Empirical research approach and data gathering mechanism

Research approach	Data gathering mechanism	Description
Expert interviews	Thai fish ecosystem qualitative, open-ended interviews (recorded and transcribed) – 13 Experts	1 Vessel owner 1 Seafood trader 2 Seafood purchasing managers (a processor and a wholesaler) 6 Managing directors of tech providers 1 General manager and auditor of the Marine Stewardship Council 2 Government officers
	Digital technologies & supply chain management qualitative, open-ended interviews – 4 Experts	4 Digital technology and supply chain managers (for validation purposes)
Field observations	Physical walkthroughs in multinational organisations	2 Fishermen in commercial fishing operations 1 Processor

For validation and verification purposes regarding the feasibility of the envisioned AI and BCT implementation framework in supply chains, we consulted four digital technology and supply chain experts with long-standing experience in digital transformation and advancements within network operations.

Empirical evidence was gathered from a single fish industry case study involving experts from different business echelons in Thailand. Notwithstanding the generalisability limitations of single case study research (Voss et al., 2002), the related focus provides unusual research access (Yin, 1993) and the range of involved informants allows the in-depth exploration of the studied phenomenon (Gobbi & Hsuan, 1995). Furthermore, single case studies are recommended to examine novel interventions such as AI and BCT.

#### Supply chain mapping

In order to understand the fish supply chain operations and the respective material, data, and information flows, we mapped the current state of the Thai fish ecosystem by combining primary and secondary evidence. We performed interviews and physical walkthroughs to observe and comprehend supply chain operations in the industry (Srai, 2017).

Understanding the current state of operations is essential to outline a future state emerging from the joint implementation of AI and BCT. To this effect, supply chain mapping was selected as an appropriate approach to capture the operations and the material/data/information flows in the Thai fish ecosystem of operations (Srai, 2017). The entailed business processes are the conceptual building blocks in the developed supply chain mapping diagrams (Holweg et al., 2018).

Considering the research scope on AI and BCT, an inherent need to ensure consistency with established information technology-based reference frameworks existed. Therefore, to ensure such consistency, we selected the BPMN as an approach to notate operations and the associated material/process/data/information flows. In particular, the OMG BPMN 2.0 was selected, which is typically used to model business processes to inform processes’ implementation. The BPMN is specified in detail by the ISO/IEC 19,510:2013 and the OMG Specification v2.0.2 (http://www.omg.org/spec/BPMN). Rosing et al. (2014) provided an operational summary of the formalisations used to develop BPMN diagrams.

#### System conceptualisation

Following empirical evidence gathering, in tandem with the literature analysis on AI and BCT implementations, we adopted the Systems Thinking view (Forrester, 1961). We used the principles of System Dynamics to depict, explicate and comprehend the interplay of these digital technologies and supply chain operations for sustainability and data monetisation (Meadows, 1980). System Dynamics has been proven successful in capturing the sustainability impact of food supply chains involving commodities such as phosphorus (El Wali et al., 2021), milk (Mangla et al., 2021) and wine (Taghikhah et al., 2021).

Digital technologies such as AI and blockchain are within the scope of System Dynamics modelling (Bhattacharyya & Nair, 2019). In addition, such digital interventions are useful for investigating the dynamics of systems like supply chains (Afanasyev et al., 2022). Therefore, we captured the structural interdependencies among AI, BCT, and supply chains through a group model building process grounded in the System Dynamics literature (Vennix, 1996). The resulting causal loop diagram is a qualitative system map that visualises the ‘AI-BCT-supply chain’ system constructs, structural elements, and interrelations. We envision that this view provides an essential, actionable framework in Operations Management.

## Fish supply chains

Fish products are essential for global food and nutritional security (FAO, 2020). Nevertheless, a range of factors may have a detrimental impact on the global supply of fish products, including: (i) growing global demand for seafood; (ii) emerging role of fish in human diets; (iii) increasing economic considerations of national and global fish trade; (iv) increasing fraudulent incidents regarding fish safety and quality; and (v) elevated consumers’ awareness about fish provenance and authenticity (Gopi et al., 2019). Therefore, interventions to improve tracking and tracing of fish supplies to prevent bad practices are required to help build consumer trust, reduce fraudulent incidents and product recalls, improve public health, and generally enhance the triple-helix of sustainability in the industry (Velez-Zuazo et al., 2021).

Technology-enabled traceability and analytics are even more prominent for the Thai fish industry, considering that the national exports of fish and fishery products are valued at approximately US$6.3 billion per year (Suwannapoom, 2021). A few years ago, the European Commission issued a yellow card to Thailand and temporarily stopped any fish imports from the country due to the magnitude of illegal, unreported, and unregulated fishing activities (European Commission, 2015). Therefore, implementing an end-to-end supply chain system for seafood tracking and tracing enabled by AI and BCT could modernise the sector and foster export opportunities.

**Fig. 3:**
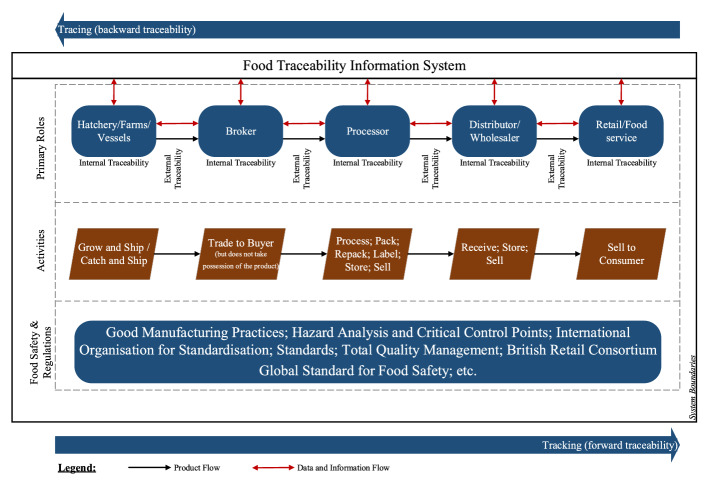
Generic fish supply chain structure and process flow *Source*: adapted from Zhang (2014)

Fish supply chains are complex ecosystems involving a plethora of stakeholders (Fig. 3). At an operational level, the main material flow across the respective supply chain is seafood. However, secondary material flows also involve various products supplied by tier-level suppliers, including packaging materials and other food ingredients. At a policy-making level, in a fish supply chain ecosystem, governmental authorities and external certification organisations, such as the Marine Stewardship Council, ISO or HACCP, are involved in the management and audit of the respective supply chain processes (Aung & Chang, 2014). The stakeholders engaged in the supply chain would have to comply with the regulations and standards set by these organisations to be allowed to involve in domestic and/or international trade activities.

### Digital technology challenges

Key technology-wise challenges in fish supply chains can be divided into three main categories: (i) inconsistencies in traceability standards; (ii) limited interoperability among the involved technology systems and databases; and (iii) limited trust among network actors.

First, it is challenging to implement a reliable full-chain traceability system in fishery supply chain ecosystems because this would require commitment and collaboration from every actor in the value chain to consistently provide reliable and accurate data and information (Global Dialogue, 2016). Many firms in seafood supply chains articulate that inconsistent global technology and traceability standards are significant obstacles to enabling full-chain traceability (Sterling et al., 2015). Quality standards and systems such as Good Manufacturing Practices, ISO 9001 or HACCP are widely accepted and implemented internationally to prevent food adulteration, product recalls, or safety hazards that may compromise consumer safety. However, these standards do not specify requirements for food supply chain traceability but rather determine operational practices during food processing; collected data is not communicated across the supply chain. Instead, regulations regarding product recalls often represent the only mandatory requirement for food traceability (Thompson et al., 2005). The requirements typically vary between countries and often provide minimal clarity on individual operators’ roles to ensure traceability or are non-existent (UK FSA, 2017). At the same time, global supply chains are positively related to product recalls due to information asymmetry between actors and lack of product traceability (Steven et al., 2014), indicating that a lack of harmonised requirements for traceability may manifest in compromised consumer safety. For example, the lack of supply chain traceability standards and associated risks became evident through high profile recall incidents such as the horsemeat scandal, which led to a tidal wave of media responses and the withdrawal of contaminated processed food. The recall occurred when food processing companies unwittingly sourced horsemeat from Eastern European suppliers and incorporated it into their products (Falkheimer et al., 2015).

Second, interoperability, i.e., “*the ability of different information technology systems and software applications to communicate, exchange data, and use the information that has been exchanged*” (HIMSS, 2013), is a critical issue in fish supply chains considering the nature of the traded commodity and the high complexity of the network. The complexity of fish supply chains is characterised by the global dispersion of the respective network and the diversity of actors involved, including fishers, buyers, processors, wholesalers, transporters, and retailers (Thompson et al., 2005). Extant research suggests that supply chain complexity exacerbates its interoperability due to the heterogeneity of interfaces and characteristics that define the supply chain (Chalyvidis et al., 2013).

Third, both industry actors’ and consumers’ limited trust is another key challenge in the sector. Data availability and information signals can be disregarded in case the data cannot be trusted. Industries depend on certification organizations such as ISO, MSC or governments to verify information related to fish supplies (Global Dialogue, 2016). However, certifications are costly, while audits are performed periodically (usually on an annual basis). In addition, investigations regarding certification practices in fish supply chains could lead to further controversy about the trustworthiness of the elaborated ecolabels (McVeigh, 2021).

### Key data elements

The quality of a traceability system, like blockchain, depends on the ability to collect and analyse necessary data from diverse sources. Different standards have different requirements, but generally, most standards, such as the EU Regulation 104/2000 and the EU Regulation 2065/2001, would require companies to record the species of origin, catch area and production method (European Commission, 2009).

Other standards, such as the TraceFish, document the necessary key data elements in fish supply chains. TraceFish standards were funded by the European Commission and coordinated by the Norwegian Institute of Fisheries and Aquaculture (Fiskeriforskning) to focus on developing a ‘Traceability of Fish Products’ (Andre, 2013). At the end of the project in 2002, CEN, the European Committee for Standardisation, published three standards that specify key data elements for fish supply chains. Particularly, TraceFish stressed the need for labelling based on unique identification numbers for all resources and end-products (Andre, 2013).

## Thai fish supply network ecosystem

The fish supply network ecosystem in Thailand involves operations at three different echelons: (i) commercial fishing; (ii) trading; and (iii) canning, which were identified as part of our ongoing research (Tsolakis et al., 2021). We excluded the local fishing operations from our analysis, despite their significant aggregated scale in terms of business operations magnitude and volume, since no data recordings are officially required by local fishermen. We further assumed that the canned fish manufacturing operations rely on the procurement of fish supplies at scale, which is feasible only via commercial fishing activities.

### Data capture and traceability

First, in terms of data capture at the commercial fishing operations echelon, the data sources refer to the Vessel Monitoring System (i.e., Global Positioning System tracker) and a vessel’s logbook. Every commercial vessel is regulated to have an installed and updated Vessel Monitoring System. Therefore, the recorded data can be deemed neither sufficient nor reliable due to: (i) the possibility of intended/unintended malfunctioning equipment; (ii) the absence of data-recording standards; and (iii) the lack of automated data gathering mechanisms. Indicatively, 20% tolerance on the weight of fish catch is allowed to fishermen by governmental regulations, while manual recordings of endangered fish species caught are not reliable. Furthermore, the fish catch is preserved in buckets that are manually filled with ice, but the cold storage temperature and fish freshness are not monitored during a vessel’s journey, which can last several days (i.e., 15–20). A photographic copy of the logbook is mandatory to be shared with arrival port authorities; however, this format does not allow the automated extraction of data related to the performed fishing activities.

Second, during trading, the unloading of the fish occurs on a metallic platform exposed to open-air environmental conditions. In addition, the weighting of the fish is performed via potentially decalibrated or malfunctioning equipment (i.e., a typical scale), while the sorting of the fish is manually performed with every sorting container receiving handwritten paper-based labelling. The pricing of the sorted fish is determined via auctions that might not reflect the actual value of the traded commodities (e.g., fish freshness, skin damages).

Third, at the manufacturing stage, the inbound tuna is manually sorted by weight and quality control inspections are based on random sampling. Downstream the processing stage, data capturing occurs systematically due to the proprietary industrial production equipment (e.g., batch number). However, the only tracing element refers to the vessel that caught the tuna, while the place of origin is not recorded.

### Artificial intelligence and blockchain in operations

Considering that this research envisions the future state of supply chain operations in the Thai fish industry enabled by AI and BCT, it is first essential to capture specific features of the fish supply network ecosystem based on the investigated businesses and comprehend unit operations performed at existing sites. Second, it is required to clarify the role of AI and BCT implementations in data and information according to the intended operational objectives and strategic commitments. Knowledge about the current state of operations helps to comprehend the underpinning material and information flows and identify key data elements that digital technologies could gather, process, share and analyse to ensure transparency, visibility, tracking and tracing. Third, the supply chain impact needs to be depicted and revised based on the global operations and business landscape developments.

**Fig. 4:**
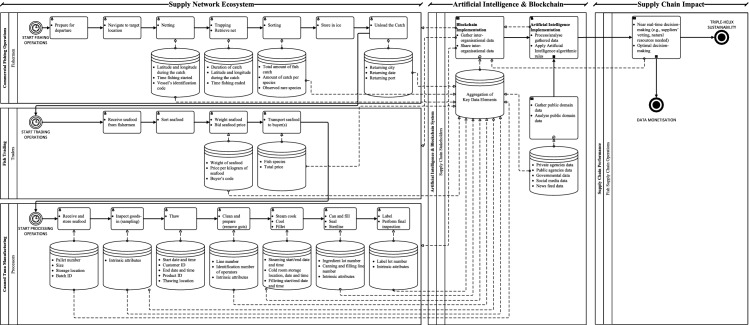
Business process map of the Thai fish supply network ecosystem enabled by Artificial Intelligence and blockchain *Source*: based on Tsolakis et al. (2021)

Therefore, we argue that the future state of fish supply chains, enabled by the joint implementation of AI and blockchain, has to be considered across three levels: (i) supply network ecosystem; (ii) AI and BCT implementation; and (iii) supply chain impact. The respective business process map of the Thai fish supply chain ecosystem enabled by AI and BCT is illustrated in Fig. 4.

At the first level, ‘Supply Network Ecosystem’, the supply chain operations are captured, including: (i) commercial fishing operations; (ii) trading; and (iii) canned tuna manufacturing (Tsolakis et al., 2021). Most importantly, key data elements that are being recorded (basically manually) are captured to inform the AI and BCT system. Although research on ‘Supply Network Ecosystems’ appears to be scarce and currently under development (Barbieri et al., 2021), applying a broader view on the supply chain related to the operations of individual actors has already shown to be an effective approach to assess sustainability in various dimensions (Hohn & Durach, 2021).

At the second level, ‘Artificial Intelligence & Blockchain’, identified key data elements can be gathered in a common database and shared across all partners via BCT. In this regard, data collected via blockchain primarily concerns inter-organisational data between upstream and downstream participants, either public or private, that actors are inclined to share and help synchronise the network operations (Wang et al., 2021). Therefore, AI can complement BCT by gathering near real-time public domain data to supplement the existing data and information flows. Considering that in the current state of operations, integrated data capture, data consistency and data interoperability archetypes are not being applied, AI algorithms could facilitate such data-related processes (Dillenberger et al., 2019). Furthermore, AI algorithms could enable automated data collection, triangulation, and validation (Kudashkina et al., 2022; Tiwari & Khan, 2019). In addition, AI algorithms may enable analyses of the data flows to detect possible disruptions (e.g., related social media posts) and inform about the agile configuration of the supply chain (e.g., identify reliable alternative suppliers) (Bottani et al., 2019).

At the third level, ‘Supply Chain Impact’, the output of AI and BCT implementations enables informed decision-making, allows accountability, and enhances collaboration and coordination across end-to-end supply chains (Rodríguez-Espíndola et al., 2020). Optimal dynamic decisions about operational aspects such as distribution and inventory management ensure improved network performance regarding service level, quality control, timeliness, and inventory position. Therefore, improvements in resources appropriation, lead times, wastage, and product recalls are achieved, ultimately leading to sustainable performance (Tsolakis et al., 2021). The improved efficiency further enables the delivery of value, both upstream and downstream of the supply chain, thus supporting competitiveness and driving data monetisation (Bechtsis et al., 2021).

### Interplay between digital technologies and supply chain operations

The complexity and non-linear behaviour of the digital-enabled fish supply network system are captured through a causal loop diagram developed based on the principles of System Dynamics. Causal loop diagrams have been successfully used as a mapping approach for capturing the macro-level interactions among essential structural elements of future state supply chains (Tsolakis & Srai, 2018).

The underpinning interrelations between the ‘AI-BCT-supply chain’ digital technology system and the supply chain system are captured through feedback loops. Feedback loops capture sequences of causes and effects transcending every loop (Georgiadis & Vlachos, 2004). Across each loop, system variables experience either an increase (represented by a positive polarity, “ + ”) or a decrease (represented by a negative polarity. “ − ”). Therefore, the entire loop, comprising of a sequence of interrelated system variables, is ultimately characterised as either reinforcing (denoted as “R”) or balancing (denoted as “B”). Based on the literature findings and our empirical research, we synthesised the interplay among all components of the ‘AI-BCT-supply chain’ system in the form of a causal loop diagram.

**Fig. 5:**
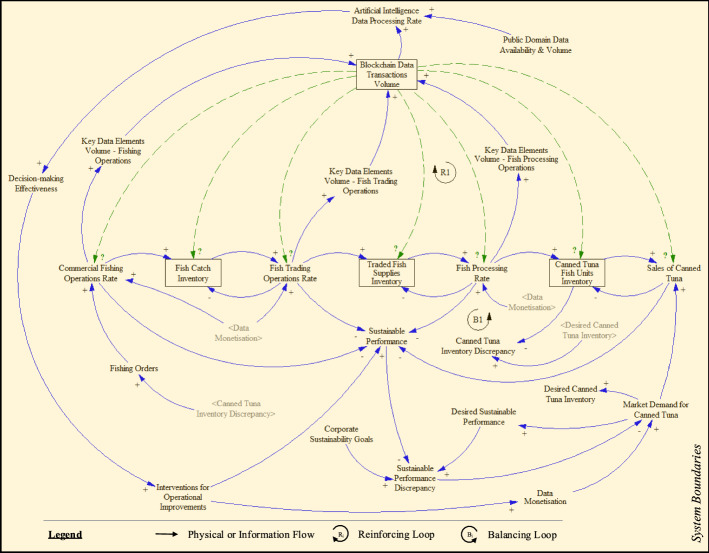
Causal loop diagram of the Thai fish supply network system enabled by Artificial Intelligence and blockchain technology

Overall, the causal loop diagram of the investigated ‘AI-BCT-supply chain’ system, validated by technology experts and operations managers, comprises 94 feedback loops (Fig. 5). The overall validation and verification process described earlier provided confidence in the relevant system considerations and the potential implications of AI and BCT implementations on the sustainability of supply chains and data monetisation. The conceptual system model was built using the Academic version of the System Dynamics simulation software Vensim^®^ PLE (× 64). However, 81 loops consider AI and BCT, of which 18 are reinforcing, and 14 are balancing. Furthermore, there are 49 pertinent loops where the effect depends on set objectives and parameters. Indicatively, in the reinforcing loop R1, the larger the ‘*Blockchain Data Transactions Volume*’, the greater the ‘*Artificial Intelligence Data Processing Rate*’ to process the available data in a timely manner. The intensity in AI-based analytics generates greater output in terms of quality that improves ‘*Decision-making Effectiveness*’. Such informed decisions then guide targeted and accurate ‘*Interventions for Operational Improvements*’ that help deliver elevated value and promote ‘*Data Monetisation*’ across all echelons of operations, such as on the ‘*Fish Processing Rate*’. Operational improvements in the respective processes necessitate the more systematic monitoring and gathering of ‘*Key Data Elements Volume—Fish Processing Operations*’, thus leading to increased ‘*Blockchain Data Transactions Volume*’.

Similarly, in the balancing loop B1, increased ‘*Blockchain Data Transactions Volume*’ requires increased ‘*Artificial Intelligence Data Processing Rate*’ for enhancing ‘*Decision-making Effectiveness*’ based on the magnitude of the generated data analysis insights. Informed decision-making then leads to targeted ‘*Interventions for Operational Improvements*’ that generate value and ‘*Data Monetisation*’ for the ‘*Fish Processing Rate*’, thus increasing the ‘*Canned Tuna Fish Units Inventory*’. However, the higher the inventory level, the lower the ‘*Canned Tuna Inventory Discrepancy*’, leading to increased ‘*Fishing Orders*’ and greater ‘*Commercial Fishing Operations Rate*’. Following that, intensified fishing operations lead to greater ‘*Key Data Elements Volume—Fishing Operations*’ that increase the ‘*Blockchain Data Transactions Volume*’. A list of the 81 feedback loops governing the system’s behaviour, enabled by AI and BCT, is inserted in Appendix 2.

Notably, the systems map demonstrates that AI and BCT implementations can have a significant role in the operational performance of food supply network systems, as 81 out of the total 94 feedback loops are based on the pertinent variables. However, the sustainability and data monetisation impact stemming from the implementation of AI and BCT depends on the objectives and parameters set by the supply chain stakeholders, as denoted by the 49 loops with unspecified polarity.

## Artificial intelligence and blockchain in supply chains: a unified framework

The design of modern and complex supply chains, specifically in the food sector, primarily involves existing data structures and technology specifications utilised by every involved stakeholder. However, data consistency, data capture, systems compatibility, data interoperability, and data architecture related issues pose significant technical barriers to the coordination and synchronisation of network operations (Tsolakis et al., 2021). Considering the limited visibility of unexpected/emergent situations and risks (e.g., Suez Canal blockage) and contemporary developments (e.g., raw material price fluctuations), supply chains could benefit from jointly adopting digital technologies such as AI and BCT.

First, the implementation of BCT, supported by an extended network of sensory infrastructure (e.g., radio-frequency identification), and AI shall be used complementarily (Babich & Hilary, 2020) to enable automated and tamper-proof data gathering and analysis. The combined implementation of digital interventions can help overcome inherent technical barriers of the supply chains, complement the capabilities of the individual technology applications, and drive sustainable performance and data monetisation, specifically within the context of volatility, uncertainty, complexity, and ambiguity in global supply chain operations (Bechtsis et al., 2021).

Second, the case study analysis results revealed that the combined implementation of AI and BCT provides multiple unique advantages to fish supply chains that lead to sustainability performance improvements. Notably, AI and BCT can enable the reduction of data gaps. This advantage results from integrating supply chain internal data streams through BCT and external data streams through AI. While BCT enables immutable data flows across nodes in end-to-end supply chains, AI supports collecting and evaluating data from external sources. Considering the improved data availability enabled by these technologies, we support that the near real-time analysis of supply chain system data and information flows enables security, operational efficiency, and productivity (Ekramifard et al., 2020), which ultimately foster sustainable performance (Pimenidis et al., 2021).

Third, our results further showed that AI enables data interoperability by overcoming sources of data inconsistency caused by supply chain complexity and diverse data recording and sharing archetypes and protocols. AI can integrate inconsistencies due to misaligned interfaces in the supply chain. Tackling inconsistencies is critical as the lack of data interoperability is still considered one of the significant barriers to BCT adoption for supply chain-related purposes (Dutta et al., 2020). Lastly, we showcased that streamlining internal and external data can help overcome an essential barrier to adopting these technologies: a lack of data monetisation for increasing economic efficiency. Data monetisation primarily stems from devising realistic market and operations scenarios and informing the decision-making process, leading to enhanced competitiveness.

**Fig. 6:**
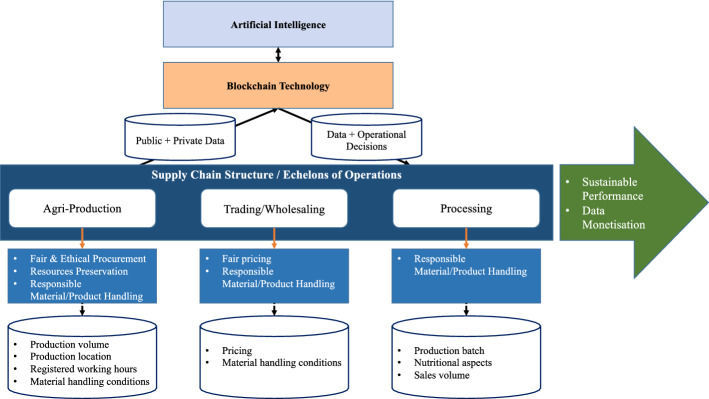
Supply chain ecosystem framework, enabled by Artificial Intelligence and blockchain, for sustainability and data monetisation

Pertaining to this efficiency, transparent and traceable production enables improvements in fair and ethical procurement, responsible handling, and conservation of resources by reducing illegal, unreported, and unregulated fishing. Demand and supply data can then be used to evaluate more efficient trading/wholesaling processes by optimising pricing whilst enabling sustainability considerations (e.g., fish freshness). In addition, a more transparent fish supply chain provides the basis for operational efficiency, for example, by improving material/product handling conditions and thus ensuring downstream health and safety of consumers. Figure 6 illustrates the method-agnostic unified framework for supply chain sustainability and data monetisation enabled by the joint implementation of AI and BCT.

## Conclusions

The digitalisation of food supply chains can help transparency and traceability while driving sustainable performance and data monetisation. As the decision-making process in food supply chains takes place on three levels, i.e., strategic, tactical, and operational (Tsolakis et al., 2014), data sharing and analysis should be performed at every echelon where the most pertinent value benefits are applicable. To a greater extent, data integration, data analysis and data/information sharing can inform the decision-making process about supply chain objectives whilst ensuring data monetisation. Our findings help respond to the enunciated research query by investigating a real-world fish supply chain and providing a business process map capturing the pertinent material, data, and information flows, followed by a systems map visualising the underpinning interrelations enabled through the joint implementation of AI and BCT in operations. The proposed unified framework summarises the key data elements that need to be recorded, shared, and analysed across the main echelons of a food supply chain as a basis for successfully implementing AI and BCT in operations.

### Academic contributions

Our research contributes to the Operations Management field by proposing a framework for the integrated implementation of AI and BCT to overcome the inherent limitations of these digital technologies. The framework implies the use of static in nature mapping tools (e.g., BPMN), while dynamic phenomena and the complex nature of the supply chain ecosystem can be captured through systems approaches (e.g., System Dynamics).

Our analysis output suggests that the processing level of data is not being captured on the blockchain, which has a significant impact on achieving sustainable goals. To this effect, the importance of data analytics, involving System Dynamics and machine learning, as a key element of BCT implementation is particularly emphasised to inform real-time decision making for sustainability and data monetisation. This particular area of information generated from diverse data sources has not been comprehensively studied in the extant literature. Furthermore, our systems map demonstrates that the behaviour of a ‘digital technology- supply chain operations’ system is mainly dictated by the applied digital technologies and the operational objectives. This observation about system interactions was revelatory to the research team and the involved experts.

Although the topic of the individual implementation of AI and BCT has been explored in the extant body of the Operations Management literature from a theoretical perspective to the best of our knowledge, this research is the first to provide an understanding of the ‘digital technology-supply chain operations’ system structure and the underpinning interplay. Moreover, the combination of business process and systems mapping provided confidence in the relevant considerations while unveiling the approach’s revelatory power.

### Practical implications

Overall, the output of the iterative mapping process led to the realisation of the following aspects of the Thai fish supply chain:

- An overarching view on key industrial/institutional actors and primary value-adding operations.
- A product perspective is emphasised through respective material flows.
- Key data elements (or data triggers) enable the tracking and tracing of fish supplies and seafood throughout end-to-end operations.
- A business process perspective indicates relevant material, data, and information flows.
- A systems perspective reveals the system interactions that dictate sustainability and data monetisation.

Specifically, the findings of our case study first highlighted the weaknesses in the fish supply chain and revealed opportunities for driving sustainability and data monetisation in fishery ecosystems. As a critical outcome of our empirical engagement with experts, we observed that neither data-sharing nor visibility mechanisms exist across the three identified levels of operations, namely: (i) commercial fishing; (ii) trading; and (iii) canning. The observed manual data capture and recording mechanisms, and communication thereof, are considered neither sufficient nor reliable for transparency, traceability, sustainability, and data monetisation purposes.

Second, it is evident that a mere analysis of the data in standardised formats, like the proposed BPMN diagram, is only the first step in introducing digital interventions. Investigating the underpinning interplay among material, data, and information flows, enabled by AI and BCT implementations, can help inform operational interventions that foster sustainability and data monetisation.

Third, the proposed unified framework captures the key data elements that need to be digitally handled in AI and BCT enabled supply chain operations in the food sector for driving value delivery. Finally, in line with the observations of Choi (2020) for the banking sector, we further argue that the combined implementation of AI and BCT can assist certification bodies in their functional role, thus possibly reducing the respective service fees.

### Limitations

This research is characterised by limitations that could motivate future research. First, the proposed framework is based on empirical evidence from a single case study of the Thai fish industry. Therefore, additional case studies in the food sector (from both developed and developing countries) need to be investigated to validate and improve the proposed framework. Further case studies will allow the generalisability of our framework whilst capturing and unveiling any additional idiosyncratic elements of the food industry. Second, in the context of our fieldwork, some fishermen hesitated to provide information on fishing methods and data records. This hesitancy may be attributed to the fact that fishermen did not implement the rapid changes in institutional regulations in a timely manner. Third, our research approach focuses on the operations-wise implications of AI and BCT implementations without delving into the technical details.

### Future research

Despite the blockchain-related benefits for supply chains, businesses are still sceptical about adopting digital technologies in operations due to consumers’ limited appreciation of the merits of AI and blockchain, and the high technical complexity and implementation cost (Kumar et al., 2019). Future studies will expand our focus from the fish supply chain to the agricultural sector in terms of system efficiency and sustainability, especially concerning limited natural and business resources such as water (Aivazidou & Tsolakis, 2021). In addition, the barriers to the implementation of AI and BCT through data privacy concerns identified in this study will motivate new research ideas for the implementation of these technologies in terms of data ownership, which will further explore the trade-off between information losses and privacy protection.

Finally, there are research opportunities for implementing AI and blockchain in industries other than the agri-food sector, such as the automotive, pharmaceutical, and aerospace industries. The proposed framework for the combined implementation of AI and BCT will ultimately need to be actively tested in a pragmatic context to investigate the impact of supply chain design, configuration, and management on the functionality of these technologies in an industrial context.

## Appendix 1

### Interview protocol

In order to conduct the semi-structured interviews, a series of questions were designed, focusing on the Key Data Elements used by the engaged Thai fish supply chain stakeholders for traceability purposes. The questions were open-ended and revolved around the issues:

- Is there an established fish traceability system across your operations?
- What is the data recording method used in your operations (e.g., paper-based, barcode, RFID)?
- What are the Key Data Elements recorded in your operations?
- How are the Key Data Elements recorded (e.g., manually, automatically)?
- Is there a data-sharing system between fish supply chain stakeholders?
- In what ways can blockchain transform the fishery industry?
- What are the key implementation challenges of digital traceability and transparency technologies in the fishery industry?

Following the interviews, data collection methods were evaluated by participating in physical walkthroughs, observing the process, and analysing whether it can be verifiable. The data collected from the case study was compared with the list of Key Data Elements recommended by TraceFish (i.e., an acceptable standard in the industry and a standard that focuses on fishery) to determine the completeness of every stakeholder’s data set. An indicative tool used to collect detailed information requirements in a structured manner from the engaged fish supply chain stakeholders is inserted in Table

**Table 4:** Detailed information requirements on Key data elements–Interview supporting tool

No.	Available	Recorded	Who recorded it?	Where did the data come from? How was it measured?	Where is it recorded?	Shall	Should	Maybe	Notes
...									

*Shall* Compulsory information, e.g., name and address of processing establishment; Should Recommended information, e.g., product temperature; *Maybe* Additional information, e.g., fish quality schemes

4.

## Appendix 2

### System dynamics model structure

Table 5 inserts a list of the 81 feedback loops governing the supply chain system’s behaviour, enabled by AI and BCT.

**Table 5:** Structure of the feedback loops of the system’s causal loop diagram.

Feedback loop	Causal effect sequence
Reinforcing, R1	Blockchain Data Transactions Volume → Artificial Intelligence Data Processing Rate → Decision-making Effectiveness → Interventions for Operational Improvements → Data Monetisation → Fish Processing Rate → Key Data Elements Volume—Fish Processing Operations → Blockchain Data Transactions Volume
Reinforcing, R2	Blockchain Data Transactions Volume → Artificial Intelligence Data Processing Rate → Decision-making Effectiveness → Interventions for Operational Improvements → Data Monetisation → Commercial Fishing Operations Rate → Key Data Elements Volume—Fishing Operations → Blockchain Data Transactions Volume
Reinforcing, R3	Blockchain Data Transactions Volume → Artificial Intelligence Data Processing Rate → Decision-making Effectiveness → Interventions for Operational Improvements → Data Monetisation → Fish Trading Operations Rate → Key Data Elements Volume—Fish Trading Operations → Blockchain Data Transactions Volume
Reinforcing, R4	Blockchain Data Transactions Volume → Artificial Intelligence Data Processing Rate → Decision-making Effectiveness → Interventions for Operational Improvements → Data Monetisation → Commercial Fishing Operations Rate → Fish Catch Inventory → Fish Trading Operations Rate → Key Data Elements Volume—Fish Trading Operations → Blockchain Data Transactions Volume
Reinforcing, R5	Blockchain Data Transactions Volume → Artificial Intelligence Data Processing Rate → Decision-making Effectiveness → Interventions for Operational Improvements → Data Monetisation → Fish Trading Operations Rate → Traded Fish Supplies Inventory → Fish Processing Rate → Key Data Elements Volume—Fish Processing Operations → Blockchain Data Transactions Volume
Reinforcing, R6	Blockchain Data Transactions Volume → Artificial Intelligence Data Processing Rate → Decision-making Effectiveness → Interventions for Operational Improvements → Data Monetisation → Commercial Fishing Operations Rate → Fish Catch Inventory → Fish Trading Operations Rate → Traded Fish Supplies Inventory → Fish Processing Rate → Key Data Elements Volume—Fish Processing Operations → Blockchain Data Transactions Volume
Reinforcing, R7	Blockchain Data Transactions Volume → Artificial Intelligence Data Processing Rate → Decision-making Effectiveness → Interventions for Operational Improvements → Data Monetisation → Market Demand for Canned Tuna → Desired Canned Tuna Inventory → Canned Tuna Inventory Discrepancy → Fishing Orders → Commercial Fishing Operations Rate → Key Data Elements Volume—Fishing Operations → Blockchain Data Transactions Volume
Reinforcing, R8	Blockchain Data Transactions Volume → Artificial Intelligence Data Processing Rate → Decision-making Effectiveness → Interventions for Operational Improvements → Data Monetisation → Market Demand for Canned Tuna → Sales of Canned Tuna → Canned Tuna Fish Units Inventory → Canned Tuna Inventory Discrepancy → Fishing Orders → Commercial Fishing Operations Rate → Key Data Elements Volume—Fishing Operations → Blockchain Data Transactions Volume
Reinforcing, R9	Blockchain Data Transactions Volume → Artificial Intelligence Data Processing Rate → Decision-making Effectiveness → Interventions for Operational Improvements → Sustainable Performance → Sustainable Performance Discrepancy → Market Demand for Canned Tuna → Desired Canned Tuna Inventory → Canned Tuna Inventory Discrepancy → Fishing Orders → Commercial Fishing Operations Rate → Key Data Elements Volume—Fishing Operations → Blockchain Data Transactions Volume
Reinforcing, R10	Blockchain Data Transactions Volume → Artificial Intelligence Data Processing Rate → Decision-making Effectiveness → Interventions for Operational Improvements → Sustainable Performance → Sustainable Performance Discrepancy → Market Demand for Canned Tuna → Sales of Canned Tuna → Canned Tuna Fish Units Inventory → Canned Tuna Inventory Discrepancy → Fishing Orders → Commercial Fishing Operations Rate → Key Data Elements Volume—Fishing Operations → Blockchain Data Transactions Volume
Reinforcing, R11	Blockchain Data Transactions Volume → Artificial Intelligence Data Processing Rate → Decision-making Effectiveness → Interventions for Operational Improvements → Data Monetisation → Market Demand for Canned Tuna → Desired Canned Tuna Inventory → Canned Tuna Inventory Discrepancy → Fishing Orders → Commercial Fishing Operations Rate → Fish Catch Inventory → Fish Trading Operations Rate → Key Data Elements Volume—Fish Trading Operations → Blockchain Data Transactions Volume
Reinforcing, R12	Blockchain Data Transactions Volume → Artificial Intelligence Data Processing Rate → Decision-making Effectiveness → Interventions for Operational Improvements → Sustainable Performance → Sustainable Performance Discrepancy → Market Demand for Canned Tuna → Desired Canned Tuna Inventory → Canned Tuna Inventory Discrepancy → Fishing Orders → Commercial Fishing Operations Rate → Fish Catch Inventory → Fish Trading Operations Rate → Key Data Elements Volume—Fish Trading Operations → Blockchain Data Transactions Volume
Reinforcing, R13	Blockchain Data Transactions Volume → Artificial Intelligence Data Processing Rate → Decision-making Effectiveness → Interventions for Operational Improvements → Data Monetisation → Market Demand for Canned Tuna → Sales of Canned Tuna → Canned Tuna Fish Units Inventory → Canned Tuna Inventory Discrepancy → Fishing Orders → Commercial Fishing Operations Rate → Fish Catch Inventory → Fish Trading Operations Rate → Key Data Elements Volume—Fish Trading Operations → Blockchain Data Transactions Volume
Reinforcing, R14	Blockchain Data Transactions Volume → Artificial Intelligence Data Processing Rate → Decision-making Effectiveness → Interventions for Operational Improvements → Sustainable Performance → Sustainable Performance Discrepancy → Market Demand for Canned Tuna → Sales of Canned Tuna → Canned Tuna Fish Units Inventory → Canned Tuna Inventory Discrepancy → Fishing Orders → Commercial Fishing Operations Rate → Fish Catch Inventory → Fish Trading Operations Rate → Key Data Elements Volume—Fish Trading Operations → Blockchain Data Transactions Volume
Reinforcing, R15	Blockchain Data Transactions Volume → Artificial Intelligence Data Processing Rate → Decision-making Effectiveness → Interventions for Operational Improvements → Data Monetisation → Market Demand for Canned Tuna → Desired Canned Tuna Inventory → Canned Tuna Inventory Discrepancy → Fishing Orders → Commercial Fishing Operations Rate → Fish Catch Inventory → Fish Trading Operations Rate → Traded Fish Supplies Inventory → Fish Processing Rate → Key Data Elements Volume—Fish Processing Operations → Blockchain Data Transactions Volume
Reinforcing, R16	Blockchain Data Transactions Volume → Artificial Intelligence Data Processing Rate → Decision-making Effectiveness → Interventions for Operational Improvements → Data Monetisation → Market Demand for Canned Tuna → Sales of Canned Tuna → Canned Tuna Fish Units Inventory → Canned Tuna Inventory Discrepancy → Fishing Orders → Commercial Fishing Operations Rate → Fish Catch Inventory → Fish Trading Operations Rate → Traded Fish Supplies Inventory → Fish Processing Rate → Key Data Elements Volume—Fish Processing Operations → Blockchain Data Transactions Volume
Reinforcing, R17	Blockchain Data Transactions Volume → Artificial Intelligence Data Processing Rate → Decision-making Effectiveness → Interventions for Operational Improvements → Sustainable Performance → Sustainable Performance Discrepancy → Market Demand for Canned Tuna → Desired Canned Tuna Inventory → Canned Tuna Inventory Discrepancy → Fishing Orders → Commercial Fishing Operations Rate → Fish Catch Inventory → Fish Trading Operations Rate → Traded Fish Supplies Inventory → Fish Processing Rate → Key Data Elements Volume—Fish Processing Operations → Blockchain Data Transactions Volume
Reinforcing, R18	Blockchain Data Transactions Volume → Artificial Intelligence Data Processing Rate → Decision-making Effectiveness → Interventions for Operational Improvements → Sustainable Performance → Sustainable Performance Discrepancy → Market Demand for Canned Tuna → Sales of Canned Tuna → Canned Tuna Fish Units Inventory → Canned Tuna Inventory Discrepancy → Fishing Orders → Commercial Fishing Operations Rate → Fish Catch Inventory → Fish Trading Operations Rate → Traded Fish Supplies Inventory → Fish Processing Rate → Key Data Elements Volume—Fish Processing Operations → Blockchain Data Transactions Volume
Balancing, B1	Blockchain Data Transactions Volume → Artificial Intelligence Data Processing Rate → Decision-making Effectiveness → Interventions for Operational Improvements → Data Monetisation → Fish Processing Rate → Canned Tuna Fish Units Inventory → Canned Tuna Inventory Discrepancy → Fishing Orders → Commercial Fishing Operations Rate → Key Data Elements Volume—Fishing Operations → Blockchain Data Transactions Volume
Balancing, B2	Blockchain Data Transactions Volume → Artificial Intelligence Data Processing Rate → Decision-making Effectiveness → Interventions for Operational Improvements → Data Monetisation → Fish Trading Operations Rate → Traded Fish Supplies Inventory → Fish Processing Rate → Canned Tuna Fish Units Inventory → Canned Tuna Inventory Discrepancy → Fishing Orders → Commercial Fishing Operations Rate → Key Data Elements Volume—Fishing Operations → Blockchain Data Transactions Volume
Balancing, B3	Blockchain Data Transactions Volume → Artificial Intelligence Data Processing Rate → Decision-making Effectiveness → Interventions for Operational Improvements → Data Monetisation → Fish Processing Rate → Canned Tuna Fish Units Inventory → Canned Tuna Inventory Discrepancy → Fishing Orders → Commercial Fishing Operations Rate → Fish Catch Inventory → Fish Trading Operations Rate → Key Data Elements Volume—Fish Trading Operations → Blockchain Data Transactions Volume
Balancing, B4	Blockchain Data Transactions Volume → Artificial Intelligence Data Processing Rate → Decision-making Effectiveness → Interventions for Operational Improvements → Data Monetisation → Fish Processing Rate → Sustainable Performance → Sustainable Performance Discrepancy → Market Demand for Canned Tuna → Desired Canned Tuna Inventory → Canned Tuna Inventory Discrepancy → Fishing Orders → Commercial Fishing Operations Rate → Key Data Elements Volume—Fishing Operations → Blockchain Data Transactions Volume
Balancing, B5	Blockchain Data Transactions Volume → Artificial Intelligence Data Processing Rate → Decision-making Effectiveness → Interventions for Operational Improvements → Data Monetisation → Fish Trading Operations Rate → Sustainable Performance → Sustainable Performance Discrepancy → Market Demand for Canned Tuna → Desired Canned Tuna Inventory → Canned Tuna Inventory Discrepancy → Fishing Orders → Commercial Fishing Operations Rate → Key Data Elements Volume—Fishing Operations → Blockchain Data Transactions Volume
Balancing, B6	Blockchain Data Transactions Volume → Artificial Intelligence Data Processing Rate → Decision-making Effectiveness → Interventions for Operational Improvements → Data Monetisation → Fish Processing Rate → Sustainable Performance → Sustainable Performance Discrepancy → Market Demand for Canned Tuna → Sales of Canned Tuna → Canned Tuna Fish Units Inventory → Canned Tuna Inventory Discrepancy → Fishing Orders → Commercial Fishing Operations Rate → Key Data Elements Volume—Fishing Operations → Blockchain Data Transactions Volume
Balancing, B7	Blockchain Data Transactions Volume → Artificial Intelligence Data Processing Rate → Decision-making Effectiveness → Interventions for Operational Improvements → Data Monetisation → Fish Trading Operations Rate → Sustainable Performance → Sustainable Performance Discrepancy → Market Demand for Canned Tuna → Sales of Canned Tuna → Canned Tuna Fish Units Inventory → Canned Tuna Inventory Discrepancy → Fishing Orders → Commercial Fishing Operations Rate → Key Data Elements Volume—Fishing Operations → Blockchain Data Transactions Volume
Balancing, B8	Blockchain Data Transactions Volume → Artificial Intelligence Data Processing Rate → Decision-making Effectiveness → Interventions for Operational Improvements → Data Monetisation → Fish Trading Operations Rate → Traded Fish Supplies Inventory → Fish Processing Rate → Sustainable Performance → Sustainable Performance Discrepancy → Market Demand for Canned Tuna → Desired Canned Tuna Inventory → Canned Tuna Inventory Discrepancy → Fishing Orders → Commercial Fishing Operations Rate → Key Data Elements Volume—Fishing Operations → Blockchain Data Transactions Volume
Balancing, B9	Blockchain Data Transactions Volume → Artificial Intelligence Data Processing Rate → Decision-making Effectiveness → Interventions for Operational Improvements → Data Monetisation → Fish Processing Rate → Canned Tuna Fish Units Inventory → Sales of Canned Tuna → Sustainable Performance → Sustainable Performance Discrepancy → Market Demand for Canned Tuna → Desired Canned Tuna Inventory → Canned Tuna Inventory Discrepancy → Fishing Orders → Commercial Fishing Operations Rate → Key Data Elements Volume—Fishing Operations → Blockchain Data Transactions Volume
Balancing, B10	Blockchain Data Transactions Volume → Artificial Intelligence Data Processing Rate → Decision-making Effectiveness → Interventions for Operational Improvements → Data Monetisation → Fish Processing Rate → Sustainable Performance → Sustainable Performance Discrepancy → Market Demand for Canned Tuna → Desired Canned Tuna Inventory → Canned Tuna Inventory Discrepancy → Fishing Orders → Commercial Fishing Operations Rate → Fish Catch Inventory → Fish Trading Operations Rate → Key Data Elements Volume—Fish Trading Operations → Blockchain Data Transactions Volume
Balancing, B11	Blockchain Data Transactions Volume → Artificial Intelligence Data Processing Rate → Decision-making Effectiveness → Interventions for Operational Improvements → Data Monetisation → Fish Processing Rate → Sustainable Performance → Sustainable Performance Discrepancy → Market Demand for Canned Tuna → Sales of Canned Tuna → Canned Tuna Fish Units Inventory → Canned Tuna Inventory Discrepancy → Fishing Orders → Commercial Fishing Operations Rate → Fish Catch Inventory → Fish Trading Operations Rate → Key Data Elements Volume—Fish Trading Operations → Blockchain Data Transactions Volume
Balancing, B12	Blockchain Data Transactions Volume → Artificial Intelligence Data Processing Rate → Decision-making Effectiveness → Interventions for Operational Improvements → Data Monetisation → Fish Trading Operations Rate → Traded Fish Supplies Inventory → Fish Processing Rate → Sustainable Performance → Sustainable Performance Discrepancy → Market Demand for Canned Tuna → Sales of Canned Tuna → Canned Tuna Fish Units Inventory → Canned Tuna Inventory Discrepancy → Fishing Orders → Commercial Fishing Operations Rate → Key Data Elements Volume—Fishing Operations → Blockchain Data Transactions Volume
Balancing, B13	Blockchain Data Transactions Volume → Artificial Intelligence Data Processing Rate → Decision-making Effectiveness → Interventions for Operational Improvements → Data Monetisation → Fish Trading Operations Rate → Traded Fish Supplies Inventory → Fish Processing Rate → Canned Tuna Fish Units Inventory → Sales of Canned Tuna → Sustainable Performance → Sustainable Performance Discrepancy → Market Demand for Canned Tuna → Desired Canned Tuna Inventory → Canned Tuna Inventory Discrepancy → Fishing Orders → Commercial Fishing Operations Rate → Key Data Elements Volume—Fishing Operations → Blockchain Data Transactions Volume
Balancing, B14	Blockchain Data Transactions Volume → Artificial Intelligence Data Processing Rate → Decision-making Effectiveness → Interventions for Operational Improvements → Data Monetisation → Fish Processing Rate → Canned Tuna Fish Units Inventory → Sales of Canned Tuna → Sustainable Performance → Sustainable Performance Discrepancy → Market Demand for Canned Tuna → Desired Canned Tuna Inventory → Canned Tuna Inventory Discrepancy → Fishing Orders → Commercial Fishing Operations Rate → Fish Catch Inventory → Fish Trading Operations Rate → Key Data Elements Volume—Fish Trading Operations → Blockchain Data Transactions Volume
Dependant, D1	Blockchain Data Transactions Volume → Commercial Fishing Operations Rate → Key Data Elements Volume—Fishing Operations → Blockchain Data Transactions Volume
Dependant, D2	Blockchain Data Transactions Volume → Fish Processing Rate → Key Data Elements Volume—Fish Processing Operations → Blockchain Data Transactions Volume
Dependant, D3	Blockchain Data Transactions Volume → Fish Trading Operations Rate → Key Data Elements Volume—Fish Trading Operations → Blockchain Data Transactions Volume
Dependant, D4	Blockchain Data Transactions Volume → Fish Catch Inventory → Fish Trading Operations Rate → Key Data Elements Volume—Fish Trading Operations → Blockchain Data Transactions Volume
Dependant, D5	Blockchain Data Transactions Volume → Traded Fish Supplies Inventory → Fish Processing Rate → Key Data Elements Volume—Fish Processing Operations → Blockchain Data Transactions Volume
Dependant, D6	Blockchain Data Transactions Volume → Fish Trading Operations Rate → Traded Fish Supplies Inventory → Fish Processing Rate → Key Data Elements Volume—Fish Processing Operations → Blockchain Data Transactions Volume
Dependant, D7	Blockchain Data Transactions Volume → Commercial Fishing Operations Rate → Fish Catch Inventory → Fish Trading Operations Rate → Key Data Elements Volume—Fish Trading Operations → Blockchain Data Transactions Volume
Dependant, D8	Blockchain Data Transactions Volume → Canned Tuna Fish Units Inventory → Canned Tuna Inventory Discrepancy → Fishing Orders → Commercial Fishing Operations Rate → Key Data Elements Volume—Fishing Operations → Blockchain Data Transactions Volume
Dependant, D9	Blockchain Data Transactions Volume → Fish Catch Inventory → Fish Trading Operations Rate → Traded Fish Supplies Inventory → Fish Processing Rate → Key Data Elements Volume—Fish Processing Operations → Blockchain Data Transactions Volume
Dependant, D10	Blockchain Data Transactions Volume → Commercial Fishing Operations Rate → Fish Catch Inventory → Fish Trading Operations Rate → Traded Fish Supplies Inventory → Fish Processing Rate → Key Data Elements Volume—Fish Processing Operations → Blockchain Data Transactions Volume
Dependant, D11	Blockchain Data Transactions Volume → Fish Processing Rate → Canned Tuna Fish Units Inventory → Canned Tuna Inventory Discrepancy → Fishing Orders → Commercial Fishing Operations Rate → Key Data Elements Volume—Fishing Operations → Blockchain Data Transactions Volume
Dependant, D12	Blockchain Data Transactions Volume → Sales of Canned Tuna → Canned Tuna Fish Units Inventory → Canned Tuna Inventory Discrepancy → Fishing Orders → Commercial Fishing Operations Rate → Key Data Elements Volume—Fishing Operations → Blockchain Data Transactions Volume
Dependant, D13	Blockchain Data Transactions Volume → Traded Fish Supplies Inventory → Fish Processing Rate → Canned Tuna Fish Units Inventory → Canned Tuna Inventory Discrepancy → Fishing Orders → Commercial Fishing Operations Rate → Key Data Elements Volume—Fishing Operations → Blockchain Data Transactions Volume
Dependant, D14	Blockchain Data Transactions Volume → Canned Tuna Fish Units Inventory → Canned Tuna Inventory Discrepancy → Fishing Orders → Commercial Fishing Operations Rate → Fish Catch Inventory → Fish Trading Operations Rate → Key Data Elements Volume—Fish Trading Operations → Blockchain Data Transactions Volume
Dependant, D15	Blockchain Data Transactions Volume → Fish Processing Rate → Canned Tuna Fish Units Inventory → Canned Tuna Inventory Discrepancy → Fishing Orders → Commercial Fishing Operations Rate → Fish Catch Inventory → Fish Trading Operations Rate → Key Data Elements Volume—Fish Trading Operations → Blockchain Data Transactions Volume
Dependant, D16	Blockchain Data Transactions Volume → Fish Trading Operations Rate → Traded Fish Supplies Inventory → Fish Processing Rate → Canned Tuna Fish Units Inventory → Canned Tuna Inventory Discrepancy → Fishing Orders → Commercial Fishing Operations Rate → Key Data Elements Volume—Fishing Operations → Blockchain Data Transactions Volume
Dependant, D17	Blockchain Data Transactions Volume → Sales of Canned Tuna → Canned Tuna Fish Units Inventory → Canned Tuna Inventory Discrepancy → Fishing Orders → Commercial Fishing Operations Rate → Fish Catch Inventory → Fish Trading Operations Rate → Key Data Elements Volume—Fish Trading Operations → Blockchain Data Transactions Volume
Dependant, D18	Blockchain Data Transactions Volume → Fish Catch Inventory → Fish Trading Operations Rate → Traded Fish Supplies Inventory → Fish Processing Rate → Canned Tuna Fish Units Inventory → Canned Tuna Inventory Discrepancy → Fishing Orders → Commercial Fishing Operations Rate → Key Data Elements Volume—Fishing Operations → Blockchain Data Transactions Volume
Dependant, D19	Blockchain Data Transactions Volume → Fish Processing Rate → Sustainable Performance → Sustainable Performance Discrepancy → Market Demand for Canned Tuna → Desired Canned Tuna Inventory → Canned Tuna Inventory Discrepancy → Fishing Orders → Commercial Fishing Operations Rate → Key Data Elements Volume—Fishing Operations → Blockchain Data Transactions Volume
Dependant, D20	Blockchain Data Transactions Volume → Traded Fish Supplies Inventory → Fish Processing Rate → Canned Tuna Fish Units Inventory → Canned Tuna Inventory Discrepancy → Fishing Orders → Commercial Fishing Operations Rate → Fish Catch Inventory → Fish Trading Operations Rate → Key Data Elements Volume—Fish Trading Operations → Blockchain Data Transactions Volume
Dependant, D21	Blockchain Data Transactions Volume → Fish Trading Operations Rate → Sustainable Performance → Sustainable Performance Discrepancy → Market Demand for Canned Tuna → Desired Canned Tuna Inventory → Canned Tuna Inventory Discrepancy → Fishing Orders → Commercial Fishing Operations Rate → Key Data Elements Volume—Fishing Operations → Blockchain Data Transactions Volume
Dependant, D22	Blockchain Data Transactions Volume → Canned Tuna Fish Units Inventory → Canned Tuna Inventory Discrepancy → Fishing Orders → Commercial Fishing Operations Rate → Fish Catch Inventory → Fish Trading Operations Rate → Traded Fish Supplies Inventory → Fish Processing Rate → Key Data Elements Volume—Fish Processing Operations → Blockchain Data Transactions Volume
Dependant, D23	Blockchain Data Transactions Volume → Sales of Canned Tuna → Sustainable Performance → Sustainable Performance Discrepancy → Market Demand for Canned Tuna → Desired Canned Tuna Inventory → Canned Tuna Inventory Discrepancy → Fishing Orders → Commercial Fishing Operations Rate → Key Data Elements Volume—Fishing Operations → Blockchain Data Transactions Volume
Dependant, D24	Blockchain Data Transactions Volume → Fish Trading Operations Rate → Sustainable Performance → Sustainable Performance Discrepancy → Market Demand for Canned Tuna → Sales of Canned Tuna → Canned Tuna Fish Units Inventory → Canned Tuna Inventory Discrepancy → Fishing Orders → Commercial Fishing Operations Rate → Key Data Elements Volume—Fishing Operations → Blockchain Data Transactions Volume
Dependant, D25	Blockchain Data Transactions Volume → Canned Tuna Fish Units Inventory → Sales of Canned Tuna → Sustainable Performance → Sustainable Performance Discrepancy → Market Demand for Canned Tuna → Desired Canned Tuna Inventory → Canned Tuna Inventory Discrepancy → Fishing Orders → Commercial Fishing Operations Rate → Key Data Elements Volume—Fishing Operations → Blockchain Data Transactions Volume
Dependant, D26	Blockchain Data Transactions Volume → Traded Fish Supplies Inventory → Fish Processing Rate → Sustainable Performance → Sustainable Performance Discrepancy → Market Demand for Canned Tuna → Desired Canned Tuna Inventory → Canned Tuna Inventory Discrepancy → Fishing Orders → Commercial Fishing Operations Rate → Key Data Elements Volume—Fishing Operations → Blockchain Data Transactions Volume
Dependant, D27	Blockchain Data Transactions Volume → Fish Processing Rate → Sustainable Performance → Sustainable Performance Discrepancy → Market Demand for Canned Tuna → Sales of Canned Tuna → Canned Tuna Fish Units Inventory → Canned Tuna Inventory Discrepancy → Fishing Orders → Commercial Fishing Operations Rate → Key Data Elements Volume—Fishing Operations → Blockchain Data Transactions Volume
Dependant, D28	Blockchain Data Transactions Volume → Sales of Canned Tuna → Canned Tuna Fish Units Inventory → Canned Tuna Inventory Discrepancy → Fishing Orders → Commercial Fishing Operations Rate → Fish Catch Inventory → Fish Trading Operations Rate → Traded Fish Supplies Inventory → Fish Processing Rate → Key Data Elements Volume—Fish Processing Operations → Blockchain Data Transactions Volume
Dependant, D29	Blockchain Data Transactions Volume → Fish Catch Inventory → Fish Trading Operations Rate → Sustainable Performance → Sustainable Performance Discrepancy → Market Demand for Canned Tuna → Desired Canned Tuna Inventory → Canned Tuna Inventory Discrepancy → Fishing Orders → Commercial Fishing Operations Rate → Key Data Elements Volume—Fishing Operations → Blockchain Data Transactions Volume
Dependant, D30	Blockchain Data Transactions Volume → Traded Fish Supplies Inventory → Fish Processing Rate → Sustainable Performance → Sustainable Performance Discrepancy → Market Demand for Canned Tuna → Sales of Canned Tuna → Canned Tuna Fish Units Inventory → Canned Tuna Inventory Discrepancy → Fishing Orders → Commercial Fishing Operations Rate → Key Data Elements Volume—Fishing Operations → Blockchain Data Transactions Volume
Dependant, D31	Blockchain Data Transactions Volume → Sales of Canned Tuna → Sustainable Performance → Sustainable Performance Discrepancy → Market Demand for Canned Tuna → Desired Canned Tuna Inventory → Canned Tuna Inventory Discrepancy → Fishing Orders → Commercial Fishing Operations Rate → Fish Catch Inventory → Fish Trading Operations Rate → Key Data Elements Volume—Fish Trading Operations → Blockchain Data Transactions Volume
Dependant, D32	Blockchain Data Transactions Volume → Fish Processing Rate → Sustainable Performance → Sustainable Performance Discrepancy → Market Demand for Canned Tuna → Desired Canned Tuna Inventory → Canned Tuna Inventory Discrepancy → Fishing Orders → Commercial Fishing Operations Rate → Fish Catch Inventory → Fish Trading Operations Rate → Key Data Elements Volume—Fish Trading Operations → Blockchain Data Transactions Volume
Dependant, D33	Blockchain Data Transactions Volume → Fish Trading Operations Rate → Traded Fish Supplies Inventory → Fish Processing Rate → Sustainable Performance → Sustainable Performance Discrepancy → Market Demand for Canned Tuna → Desired Canned Tuna Inventory → Canned Tuna Inventory Discrepancy → Fishing Orders → Commercial Fishing Operations Rate → Key Data Elements Volume—Fishing Operations → Blockchain Data Transactions Volume
Dependant, D34	Blockchain Data Transactions Volume → Fish Catch Inventory → Fish Trading Operations Rate → Sustainable Performance → Sustainable Performance Discrepancy → Market Demand for Canned Tuna → Sales of Canned Tuna → Canned Tuna Fish Units Inventory → Canned Tuna Inventory Discrepancy → Fishing Orders → Commercial Fishing Operations Rate → Key Data Elements Volume—Fishing Operations → Blockchain Data Transactions Volume
Dependant, D35	Blockchain Data Transactions Volume → Fish Processing Rate → Canned Tuna Fish Units Inventory → Sales of Canned Tuna → Sustainable Performance → Sustainable Performance Discrepancy → Market Demand for Canned Tuna → Desired Canned Tuna Inventory → Canned Tuna Inventory Discrepancy → Fishing Orders → Commercial Fishing Operations Rate → Key Data Elements Volume—Fishing Operations → Blockchain Data Transactions Volume
Dependant, D36	Blockchain Data Transactions Volume → Traded Fish Supplies Inventory → Fish Processing Rate → Canned Tuna Fish Units Inventory → Sales of Canned Tuna → Sustainable Performance → Sustainable Performance Discrepancy → Market Demand for Canned Tuna → Desired Canned Tuna Inventory → Canned Tuna Inventory Discrepancy → Fishing Orders → Commercial Fishing Operations Rate → Key Data Elements Volume—Fishing Operations → Blockchain Data Transactions Volume
Dependant, D37	Blockchain Data Transactions Volume → Fish Trading Operations Rate → Traded Fish Supplies Inventory → Fish Processing Rate → Sustainable Performance → Sustainable Performance Discrepancy → Market Demand for Canned Tuna → Sales of Canned Tuna → Canned Tuna Fish Units Inventory → Canned Tuna Inventory Discrepancy → Fishing Orders → Commercial Fishing Operations Rate → Key Data Elements Volume—Fishing Operations → Blockchain Data Transactions Volume
Dependant, D38	Blockchain Data Transactions Volume → Fish Processing Rate → Sustainable Performance → Sustainable Performance Discrepancy → Market Demand for Canned Tuna → Sales of Canned Tuna → Canned Tuna Fish Units Inventory → Canned Tuna Inventory Discrepancy → Fishing Orders → Commercial Fishing Operations Rate → Fish Catch Inventory → Fish Trading Operations Rate → Key Data Elements Volume—Fish Trading Operations → Blockchain Data Transactions Volume
Dependant, D39	Blockchain Data Transactions Volume → Canned Tuna Fish Units Inventory → Sales of Canned Tuna → Sustainable Performance → Sustainable Performance Discrepancy → Market Demand for Canned Tuna → Desired Canned Tuna Inventory → Canned Tuna Inventory Discrepancy → Fishing Orders → Commercial Fishing Operations Rate → Fish Catch Inventory → Fish Trading Operations Rate → Key Data Elements Volume—Fish Trading Operations → Blockchain Data Transactions Volume
Dependant, D40	Blockchain Data Transactions Volume → Fish Catch Inventory → Fish Trading Operations Rate → Traded Fish Supplies Inventory → Fish Processing Rate → Sustainable Performance → Sustainable Performance Discrepancy → Market Demand for Canned Tuna → Desired Canned Tuna Inventory → Canned Tuna Inventory Discrepancy → Fishing Orders → Commercial Fishing Operations Rate → Key Data Elements Volume—Fishing Operations → Blockchain Data Transactions Volume
Dependant, D41	Blockchain Data Transactions Volume → Traded Fish Supplies Inventory → Fish Processing Rate → Sustainable Performance → Sustainable Performance Discrepancy → Market Demand for Canned Tuna → Desired Canned Tuna Inventory → Canned Tuna Inventory Discrepancy → Fishing Orders → Commercial Fishing Operations Rate → Fish Catch Inventory → Fish Trading Operations Rate → Key Data Elements Volume—Fish Trading Operations → Blockchain Data Transactions Volume
Dependant, D42	Blockchain Data Transactions Volume → Traded Fish Supplies Inventory → Fish Processing Rate → Sustainable Performance → Sustainable Performance Discrepancy → Market Demand for Canned Tuna → Sales of Canned Tuna → Canned Tuna Fish Units Inventory → Canned Tuna Inventory Discrepancy → Fishing Orders → Commercial Fishing Operations Rate → Fish Catch Inventory → Fish Trading Operations Rate → Key Data Elements Volume—Fish Trading Operations → Blockchain Data Transactions Volume
Dependant, D43	Blockchain Data Transactions Volume → Fish Processing Rate → Canned Tuna Fish Units Inventory → Sales of Canned Tuna → Sustainable Performance → Sustainable Performance Discrepancy → Market Demand for Canned Tuna → Desired Canned Tuna Inventory → Canned Tuna Inventory Discrepancy → Fishing Orders → Commercial Fishing Operations Rate → Fish Catch Inventory → Fish Trading Operations Rate → Key Data Elements Volume—Fish Trading Operations → Blockchain Data Transactions Volume
Dependant, D44	Blockchain Data Transactions Volume → Fish Catch Inventory → Fish Trading Operations Rate → Traded Fish Supplies Inventory → Fish Processing Rate → Sustainable Performance → Sustainable Performance Discrepancy → Market Demand for Canned Tuna → Sales of Canned Tuna → Canned Tuna Fish Units Inventory → Canned Tuna Inventory Discrepancy → Fishing Orders → Commercial Fishing Operations Rate → Key Data Elements Volume—Fishing Operations → Blockchain Data Transactions Volume
Dependant, D45	Blockchain Data Transactions Volume → Sales of Canned Tuna → Sustainable Performance → Sustainable Performance Discrepancy → Market Demand for Canned Tuna → Desired Canned Tuna Inventory → Canned Tuna Inventory Discrepancy → Fishing Orders → Commercial Fishing Operations Rate → Fish Catch Inventory → Fish Trading Operations Rate → Traded Fish Supplies Inventory → Fish Processing Rate → Key Data Elements Volume—Fish Processing Operations → Blockchain Data Transactions Volume
Dependant, D46	Blockchain Data Transactions Volume → Fish Trading Operations Rate → Traded Fish Supplies Inventory → Fish Processing Rate → Canned Tuna Fish Units Inventory → Sales of Canned Tuna → Sustainable Performance → Sustainable Performance Discrepancy → Market Demand for Canned Tuna → Desired Canned Tuna Inventory → Canned Tuna Inventory Discrepancy → Fishing Orders → Commercial Fishing Operations Rate → Key Data Elements Volume—Fishing Operations → Blockchain Data Transactions Volume
Dependant, D47	Blockchain Data Transactions Volume → Traded Fish Supplies Inventory → Fish Processing Rate → Canned Tuna Fish Units Inventory → Sales of Canned Tuna → Sustainable Performance → Sustainable Performance Discrepancy → Market Demand for Canned Tuna → Desired Canned Tuna Inventory → Canned Tuna Inventory Discrepancy → Fishing Orders → Commercial Fishing Operations Rate → Fish Catch Inventory → Fish Trading Operations Rate → Key Data Elements Volume—Fish Trading Operations → Blockchain Data Transactions Volume
Dependant, D48	Blockchain Data Transactions Volume → Canned Tuna Fish Units Inventory → Sales of Canned Tuna → Sustainable Performance → Sustainable Performance Discrepancy → Market Demand for Canned Tuna → Desired Canned Tuna Inventory → Canned Tuna Inventory Discrepancy → Fishing Orders → Commercial Fishing Operations Rate → Fish Catch Inventory → Fish Trading Operations Rate → Traded Fish Supplies Inventory → Fish Processing Rate → Key Data Elements Volume—Fish Processing Operations → Blockchain Data Transactions Volume
Dependant, D49	Blockchain Data Transactions Volume → Fish Catch Inventory → Fish Trading Operations Rate → Traded Fish Supplies Inventory → Fish Processing Rate → Canned Tuna Fish Units Inventory → Sales of Canned Tuna → Sustainable Performance → Sustainable Performance Discrepancy → Market Demand for Canned Tuna → Desired Canned Tuna Inventory → Canned Tuna Inventory Discrepancy → Fishing Orders → Commercial Fishing Operations Rate → Key Data Elements Volume—Fishing Operations → Blockchain Data Transactions Volume
